# Biocatalytic cascade to polysaccharide amination

**DOI:** 10.1186/s13068-024-02477-6

**Published:** 2024-02-27

**Authors:** Xuebin Feng, Siyi Hong, Hongbo Zhao, Thu V. Vuong, Emma R. Master

**Affiliations:** 1 Department of Chemical Engineering and Applied Chemistry, University of Toronto, 200 College Street, Toronto, ON M5S 3E5 Canada; 2 Department of Food and Nutrition, University of Helsinki, Helsinki, Finland; 3 Department of Bioproducts and Biosystems, Aalto University, Espoo, Finland

**Keywords:** Amine transaminases, Aminated polysaccharide, Enzymatic cascade, Transaminase activity assay

## Abstract

**Background:**

Chitin, the main form of aminated polysaccharide in nature, is a biocompatible, polycationic, and antimicrobial biopolymer used extensively in industrial processes. Despite the abundance of chitin, applications thereof are hampered by difficulties in feedstock harvesting and limited structural versatility. To address these problems, we proposed a two-step cascade employing carbohydrate oxidoreductases and amine transaminases for plant polysaccharide aminations via one-pot reactions. Using a galactose oxidase from *Fusarium graminearum* for oxidation, this study compared the performance of CvATA (from *Chromobacterium violaceum*) and SpATA (from *Silicibacter pomeroyi*) on a range of oxidized carbohydrates with various structures and sizes. Using a rational enzyme engineering approach, four point mutations were introduced on the SpATA surface, and their effects on enzyme activity were evaluated.

**Results:**

Herein, a quantitative colorimetric assay was developed to enable simple and accurate time-course measurement of the yield of transamination reactions. With higher operational stability, SpATA produced higher product yields in 36 h reactions despite its lower initial activity. Successful amination of oxidized galactomannan by SpATA was confirmed using a deuterium labeling method; higher aminated carbohydrate yields achieved with SpATA compared to CvATA were verified using HPLC and XPS. By balancing the oxidase and transaminase loadings, improved operating conditions were identified where the side product formation was largely suppressed without negatively impacting the product yield. SpATA mutants with multiple alanine substitutions besides E407A showed improved product yield. The E407A mutation reduced SpATA activity substantially, supporting its predicted role in maintaining the dimeric enzyme structure.

**Conclusions:**

Using oxidase–amine transaminase cascades, the study demonstrated a fully enzymatic route to polysaccharide amination. Although the activity of SpATA may be further improved via enzyme engineering, the low operational stability of characterized amine transaminases, as a result of low retention of PMP cofactors, was identified as a key factor limiting the yield of the designed cascade. To increase the process feasibility, future efforts to engineer improved SpATA variants should focus on improving the cofactor affinity, and thus the operational stability of the enzyme.

**Graphical Abstract:**

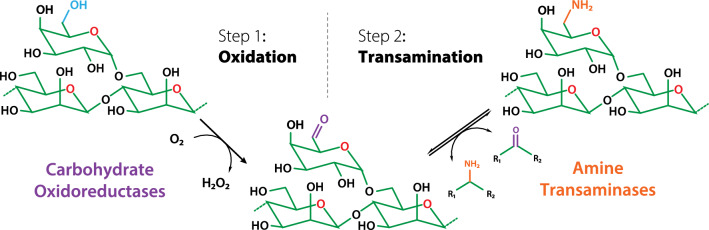

**Supplementary Information:**

The online version contains supplementary material available at 10.1186/s13068-024-02477-6.

## Background

Natural aminated polysaccharides mainly occur in forms of chitin and peptidoglycan. Whereas chitin comprises repeating *N*-acetylglucosamine, peptidoglycan consists of alternating *N*-acetylglucosamine and *N*-acetylmuramic acid; in both cases, the repeating units are connected via β-(1→4)-linkages. Chitin is a major component of arthropod exoskeletons and fungal cell walls; today, chitin extracted from crustacean shells serves as the main source of aminated polysaccharides for applied purposes [1]. In industrial processing, chitin is extracted chemically using acid–base treatments or enzymatically by proteases; it is then often deacetylated to chitosan to introduce primary amine functionalities [1, 2]. Given its biocompatible, polycationic, and antimicrobial properties, chitosan has been used as a flocculant in wastewater treatment, an enhancer in plant growth regulation, and a component of controlled-release drug matrices [3, 4]. In consumer products, chitosan has been used increasingly as a food additive, a beverage stabilizer, and a cosmetic ingredient [4]. The intensive processing requirements and challenges relating to feedstock collection from largely animal sources, however, limit the supply of sustainably sourced chitosan for the wide range of aminated polysaccharide applications. From a performance perspective, the narrow pH range conducive to solubilizing chitosan in water (pH 4.5–5) and the low mucoadhesiveness of chitosan at physiological temperature and pH further restrict its applications as a bio-flocculant for water purification or a component of biomedical materials used in controlled drug release systems [5, 6]. Compared to chitin, large-scale, diverse, and distributed processes exist that sustainably harvest plant polysaccharides, mainly cellulose, for paper, packaging, and textile manufacturing [7, 8]. Hemicelluloses, which represent a comparatively underused fraction of plant fiber, adopt a range of structures with different chemical and physical properties [9]. Although hemicelluloses and other plant polysaccharides lack amine functionality, sustainable routes to their amination would generate a plant-based alternative to chitin that also benefits from existing large-scale biorefinery processes.

**Fig. 1:**
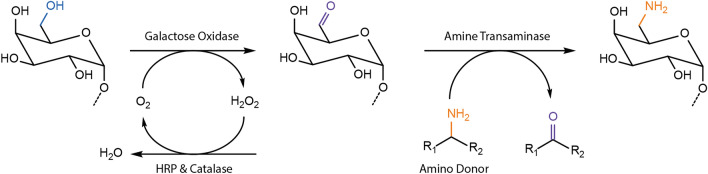
The proposed oxidation–transamination biocatalytic cascade adopted from Aumala et al. [13]. The amination of d-galactose at the C-6 position is shown as an example. Aminations of other monosaccharides and positions are possible in theory by selecting different carbohydrate oxidoreductases

Existing pathways to aminated polysaccharides from plant polysaccharides consist of mainly cellulose amination via (2,2,6,6-tetramethylpiperidin-1-yl)oxyl or periodate oxidation followed by reductive amination [5, 10–12]. Besides the use of strong oxidizers and toxic metal catalysts, over-oxidation and depolymerization of cellulose are major drawbacks of these chemical processes. Enzymatic reactions are typically performed at milder conditions and have higher specificity, and thus represent compelling alternative approaches for plant polysaccharide amination. Previously, Aumala et al. [13] demonstrated the use of a two-step cascade employing carbohydrate oxidoreductases and amine transaminases for plant carbohydrate aminations (Fig. 1): in the first step, a galactose oxidase from *Fusarium graminearum* (FgrGaOx; UniProt ID: A0A2H3HJK8) is used to introduce an aldehyde group at the C-6 hydroxyl position of galactose and galactose-containing oligosaccharides. In the second step, an amine transaminase from *Chromobacterium violaceum* (CvATA; UniProt ID: Q7NWG4) is used to transfer a primary amino group from donor molecule to the C-6 aldehyde present in the carbohydrate acceptor [13]. Briefly, galactose oxidases are copper-dependent enzymes that belong to the auxiliary activity family 5 subfamily 2 (AA5_2) in the carbohydrate-active enzyme (CAZy) database [14]. The specificity of galactose oxidase towards the C-6 hydroxyl of galactose and terminal galactose residues in oligo- and polysaccharides is well documented [15, 16], which has been exploited in productions of thermally stable hydrogels and aerogels from guar galactomannan and tamarind galactoxyloglucan [17, 18].

Transaminases (TAs), on the other hand, are pyridoxal-5′-phosphate (PLP) dependent enzymes that catalyze the transfer of primary amino groups from amines to the carbonyl carbons of ketones or aldehydes [19]. The reaction is carried out via a ping-pong mechanism in which PLP is used as an electron shuttle. First, a primary amino group from the amino donor is transferred to a PLP cofactor, which deaminates the donor and forms a pyridoxamine-5′-phosphate (PMP) intermediate; the PMP in turn donates the amino group to the acceptor, generating a target amine and recovering PLP for the next catalytic cycle [20, 21]. As members of fold types I and IV PLP-dependent enzymes, TAs are further categorized into α-TAs and amine transaminases (ATAs) based on their regioselectivity. Whereas α-TAs only catalyze the transamination between α-amino- and α-aldo/keto-acids, ATAs can in principle convert any primary amines to ketones or aldehydes (or vice versa) regardless of the location of the amino group [20]. As a result, ATAs are frequently used in asymmetric syntheses of chiral amines, which are fundamental building blocks of many high-value chemicals and pharmaceuticals [22]. A prominent success of industrial ATA applications is the synthesis of the antidiabetic (*R*)-sitagliptin using the extensively engineered ATA-117 [23]. Following a “substrate-walking” process, the active site of the ATA was expanded stepwise to accommodate bulky side groups. Compared to the chemical approach, the enzymatic process eliminates the use of toxic rhodium catalysts and improves the optical purity of the product to over 99.95% [23]. Similar processes have been developed for the production of other active pharmaceutical ingredients including norephedrine, rivastigmine, and perindopril [22]. Despite the successes in engineering ATAs for bulky monomeric amine synthesis, the activity and specificity of ATAs towards carbohydrates, including polysaccharides, remain unexplored.

Herein, CvATA and SpATA from *Silicibacter pomeroyi* (UniProt: Q5LMU1) were compared for the ability to aminate plant polysaccharides, specifically galactomannan and xyloglucan. CvATA is one of the most reported ATAs and is known for its high catalytic efficiency and broad substrate scope [24]. The enzyme was previously shown to be active on oxidized mono- and oligosaccharide substrates [13], however, its activity towards higher molecular weight oxidized polysaccharides is unknown. Previous studies showed that SpATA can accept bulky substrates including Jeffamines and halogenated prochiral ketones [25, 26], which makes it a promising enzyme for polysaccharide amination. In this study, a new colorimetric assay was established to compare the yield of aminated polysaccharides produced by CvATA and SpATA. The FgrGaOx and ATA activity in simultaneous cascades were balanced to suppress gel formation that reduces product yield by consuming oxidized intermediates. The possibility of increasing SpATA activity on polysaccharides via site-directed mutagenesis was also explored, which revealed combinations of alanine substitutions with beneficial effects on the SpATA activity towards polysaccharide substrates.

## Results

### Colorimetric assays to evaluate ATA activity on oxidized carbohydrates

The initial activity of CvATA and SpATA on FgrGaOx-oxidized galactose, lactose, melibiose, raffinose, galactomannan, and xyloglucan was measured using the acetophenone assay; transaminase activity on pyruvate was measured for comparison. Similar to the values reported by Aumala et al. [13], the initial activity of CvATA on pyruvate and oxidized galactose was 7500 ± 430 U/g and 74.2 ± 2.1 U/g, respectively. Notably, the initial activity of SpATA was roughly half that measured for CvATA, specifically 2830 ± 170 U/g and 40.5 ± 1.8 U/g on pyruvate and oxidized galactose, respectively. No significant activity was measured for either ATA on oxidized oligo- and polysaccharide substrates, likely due to the rapid evaporation of acetophenone under standard assay conditions.

**Fig. 2:**
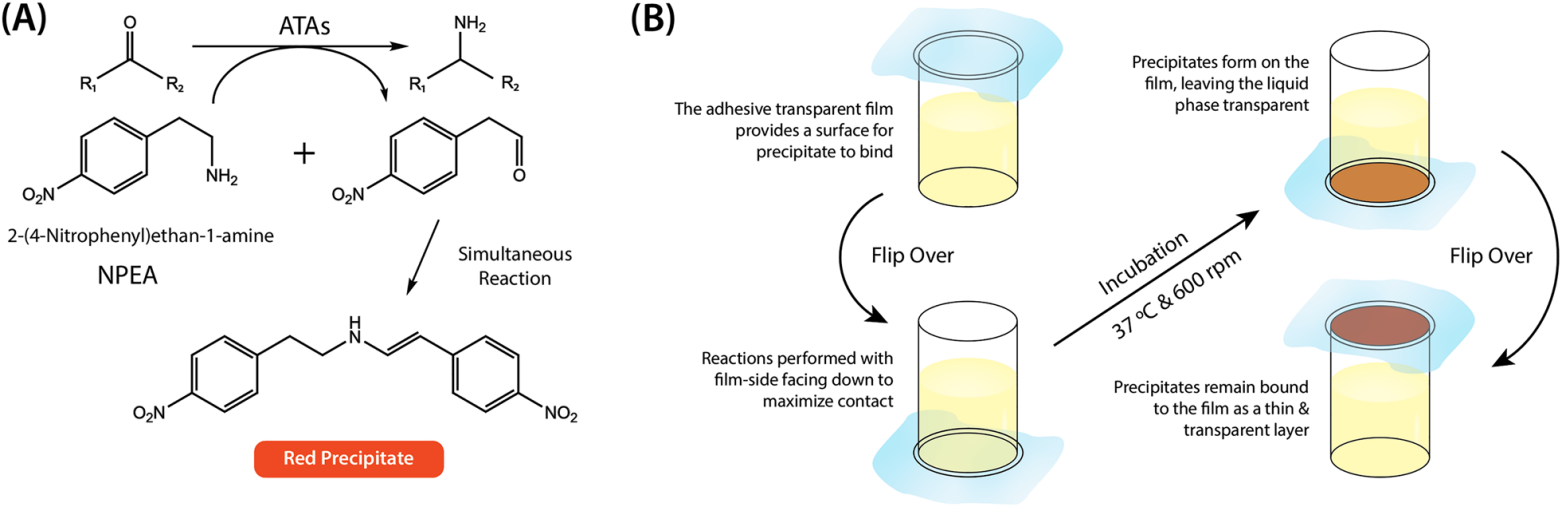
**A** Mechanism of red precipitate formation in the NPEA assay and the Q-NPEA assay. **B** Workflow of the Q-NPEA assay when performed in wells of a transparent microtiter plate

**Fig. 3:**
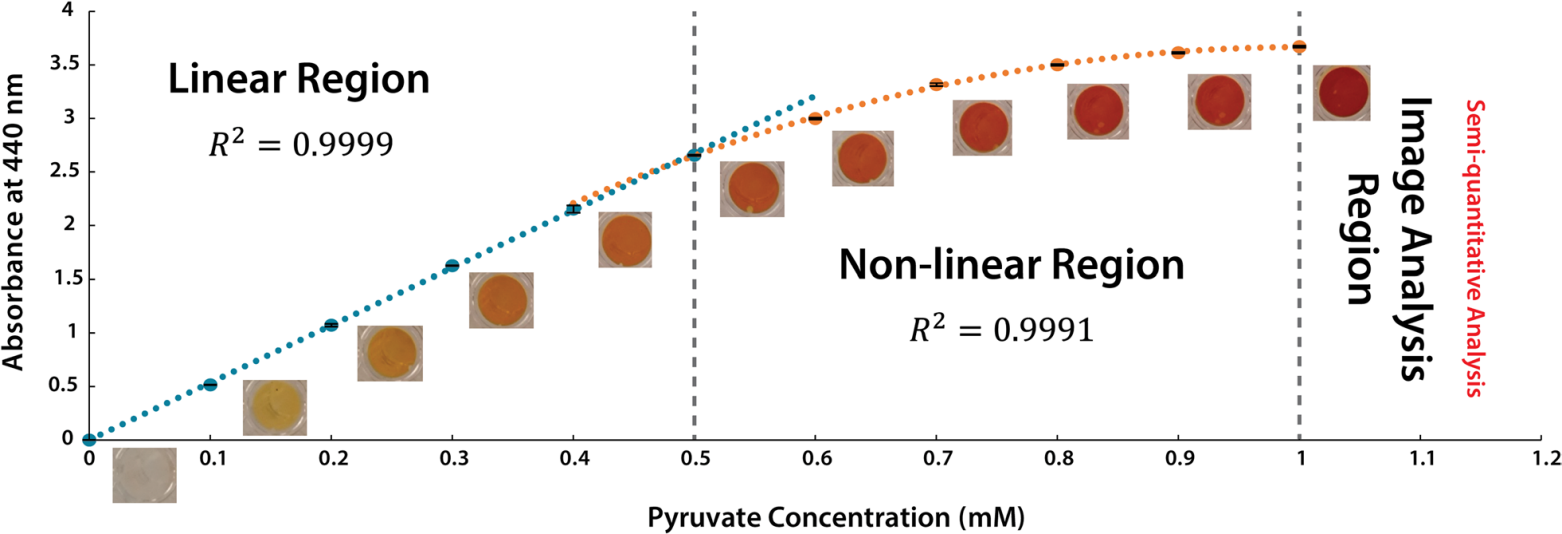
The standard curve established in this study to convert optical absorbance at 440 nm to NPEA depletion. Pyruvate with concentrations from 0.1 to 1.0 mM was used as the amino acceptor, and 10 mM NPEA was used as the amino donor. The microtiter plate was sealed with transparent and adhesive film and was incubated at 37 °C and 600 rpm for 24 h with the film side facing down. The absorbance of reaction wells was measured at 440 nm using a spectrophotometer, and depletion of pyruvate in all reactions was confirmed using HPLC-RI (Additional file 1: Fig. S4) (*n* = 4, error bars indicate standard errors)

The volatility of acetophenone and background interference that increases over time meant that the acetophenone assay is not suitable for measuring ATA-catalyzed aminations of oxidized oligo- and polysaccharides (Additional file 1: Figs. S1, S2). Instead, a variation of the 2-(4-nitrophenyl)ethan-1-amine assay (the NPEA assay), originally developed by Baud et al. [27], was developed herein to establish a colorimetric method for quantifying transamination reactions on oxidized carbohydrates. In the NPEA assay, a red precipitate forms when the NPEA reacts with its deaminated form (Fig. 2A), which is a by-product of the transamination reaction. The quantitative NPEA assay (the Q-NPEA assay) established here uses adhesive and transparent film to seal each reaction well on a microtiter plate and incubates the reactions with the film side facing down (Fig. 2B). As the reaction progresses, the red precipitate adheres to the film, forming a transparent colored layer with a maximum absorbance near 440 nm (Additional file 1: Fig. S3). The Q-NPEA assay is quantitative for amino acceptor conversions up to 1.0 mM under the experimental setup used in this study (Fig. 3); notably, the linear range can be adjusted by varying the reaction volume or the area of the adhesive surface. The accuracy of the standard curve was verified by parallel HPLC-RI measurements that quantified the depletion of pyruvate in ATA reactions comprising excess NPEA; complete depletion of 0.1 mM to 1.0 mM pyruvate was confirmed for all reactions (Additional file 1: Fig. S4).

**Fig. 4:**
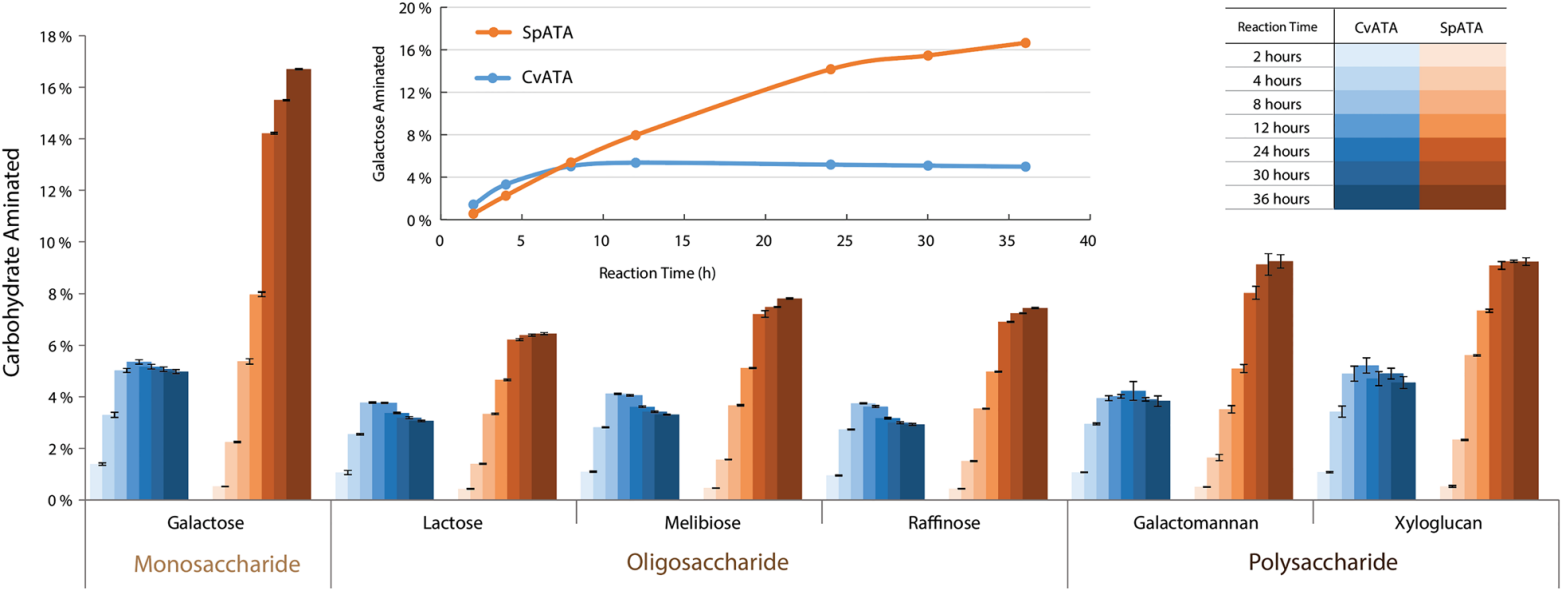
Conversions of FgrGaOx-oxidized carbohydrates through amination by CvATA (blue) and SpATA (orange) measured using the Q-NPEA assay. The oxidation and transamination reactions were performed simultaneously in one pot. The inserted line graph shows the amination of oxidized galactose with a linear time scale to underscore the higher initial activity of CvATA. Reactions (200 µL) comprised 50 mM HEPES buffer (pH 7.5), 29.8 µg/mL FgrGaOx, 12.8 µg/mL catalase, 1.8 µg/mL HRP, 150 µg/mL ATA, 20 µM PLP, 10 mM NPEA and carbohydrates containing 5 mM galactose and were carried out at 37 °C and 600 rpm. (*n* = 4, error bars indicate standard errors)

**Fig. 5:**
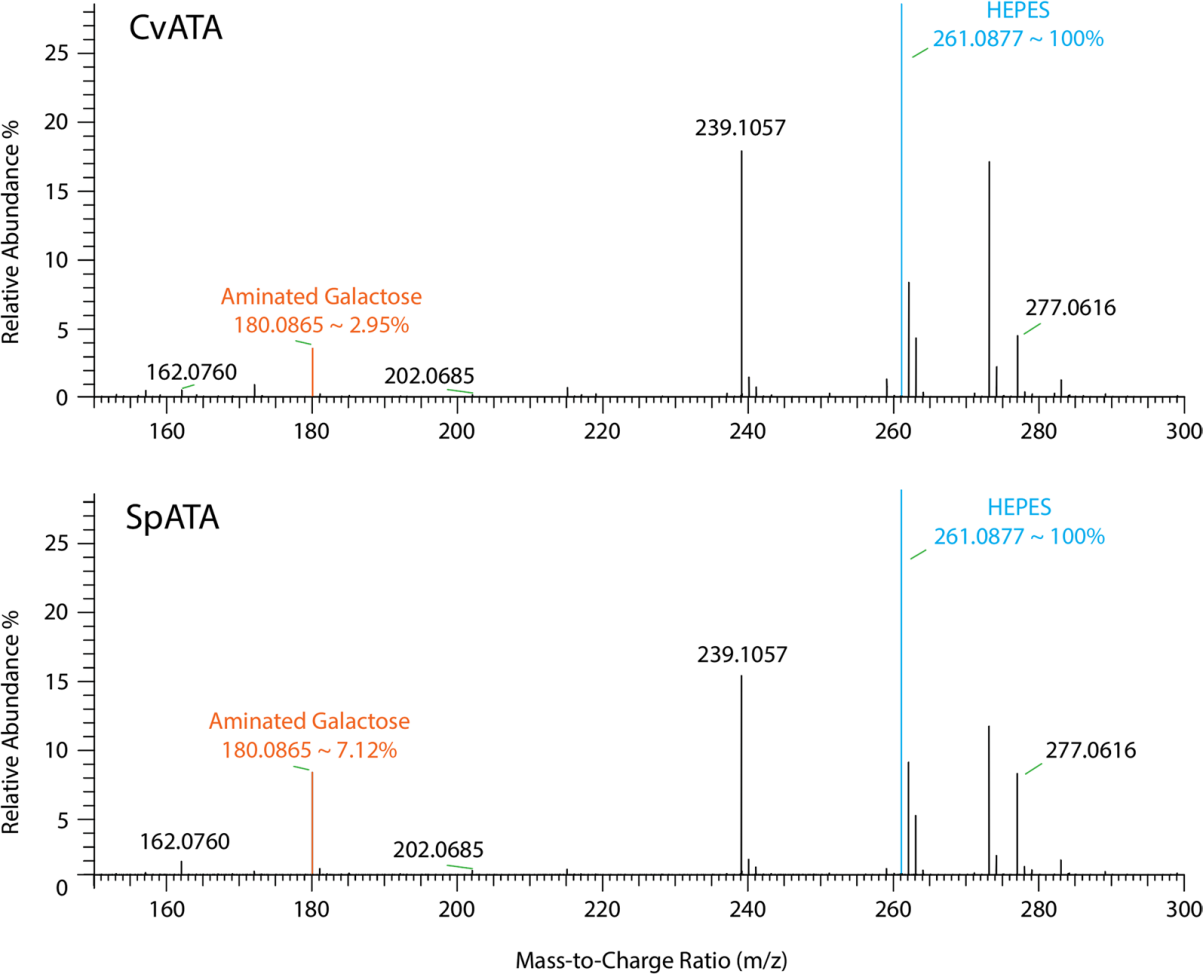
ESI-Q-TOF MS spectra (positive ionization mode) of aminated galactose produced via CvATA and SpATA treatments with simultaneous FgrGaOx oxidation. The relative abundance of aminated galactose was quantified using HEPES as an internal reference. Reactions (1 mL) comprised 50 mM HEPES (pH 7.5), 29.8 µg/mL FgrGaOx, 1.8 µg/mL HRP, 12.8 µg/mL catalase, 0.15 mg/mL ATA, 20 µM PLP, 5 mM (*S*)-1-phenylethylamine and 5 mM galactose were carried out at 37 °C and 600 rpm for 5 h

CvATA and SpATA-catalyzed transamination of FgrGaOx-oxidized oligo- and polysaccharides were then compared using the Q-NPEA assay (Fig. 4). Both ATAs were active on all tested oxidized carbohydrates despite differences in the degree of polymerization and the glycosidic linkage connecting the terminal oxidized galactose to the rest of the substrate. After 36 h, reactions comprising SpATA consistently produced higher product yield compared to those comprising CvATA, even though the latter had higher initial activity on pyruvate and oxidized galactose. The same trend was observed when using ESI-Q-TOF MS to evaluate galactose amination after 24 h, which showed the relative intensity of aminated galactose produced by SpATA was approximately twice that produced by CvATA (Fig. 5). Notably, CvATA treatments led to higher substrate conversions in the first 4 h; however, the enzyme was completely deactivated within 12 h. By contrast, SpATA remained active over the full incubation period (36 h), explaining overall higher product yields in corresponding reactions.

### Direct detection of aminated galactomannan by X-ray photoelectron spectroscopy and mass spectrometry

**Fig. 6:**
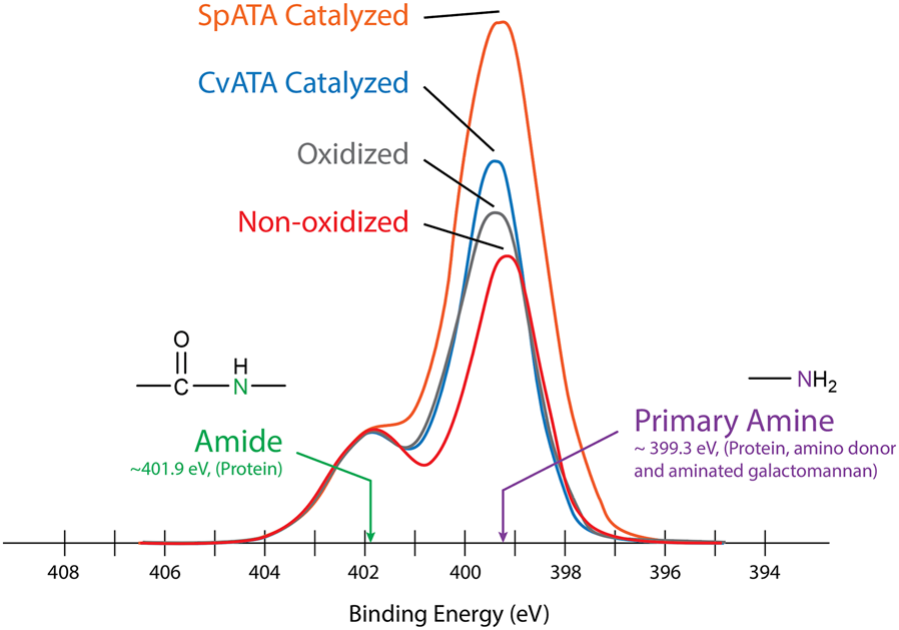
The XPS spectra showing nitrogen profiles of CvATA and SpATA aminated galactomannan with oxidized and non-oxidized galactomannan as references. The spectra were adjusted by scaling amide peaks to the same height so the amine peaks could be directly compared. The intensity of peaks is shown using a linear scale. Reactions (500 µL) comprised 50 mM potassium phosphate buffer (pH 7.5), 20 µM PLP, 29.8 µg/mL FgrGaOx, 12.8 µg/mL catalase, 1.8 µg/mL HRP, 0.15 mg/mL ATA, 5 mM (*S*)-1-PEA and 0.29% galactomannan containing 5 mM galactose. Reactions were carried out at 37 °C and 600 rpm for 24 h in 1.5 mL centrifuge tubes. The oxidized reference did not contain ATA, and the non-oxidized reference did not contain any enzymes. Enzymes (SpATA, FgrGaOx, catalase, and HRP) were added to both references after reactions to the concentrations stated above. Samples were desalted immediately using 100 kDa centrifuge filters before freeze–drying and XPS analyses

X-ray photoelectron spectroscopy (XPS) was used to detect the enzymatic introduction of primary amino groups to the oxidized polysaccharide amino acceptor, specifically oxidized galactomannan. Using the nitrogen signal from the oxidized galactomannan as a baseline, the signal corresponding to the primary amino group was 2.5 times higher after treatment with SpATA compared to CvATA (Fig. 6). To quantify the degree of amination of each product on the basis of oxidized galactose, the GC–MS-based deuterium labeling method described in Parikka et al. [15] was used to determine the percentage of oxidized galactose in galactomannan that remained after amination by each ATA. Relative to the untreated control, the degree of oxidation (DO) of the oxidized, CvATA aminated and SpATA aminated galactomannan were 54.1 ± 1.7%, 55.4 ± 1.7%, and 44.4 ± 5.9%, respectively. The reduction in DO after SpATA treatment corresponds to 9.7 ± 3.5% amination of galactose in galactomannan, or 17.9 ± 3.9% amination of the oxidized galactose subunits, which quantitatively agrees with the Q-NPEA assay result. In contrast, CvATA treatment did not produce a measurable reduction in DO, further demonstrating the low performance of CvATA on oxidized polysaccharides.

### Balancing oxidase and ATA activity to reduce gel formation

Gel formation is often observed after polysaccharide oxidation and results from acetal bond formation between aldehyde groups introduced by oxidation and hydroxyl groups abundant in the polysaccharide substrate [17]. Gelation is expected to reduce amination by reducing the available carbonyls and imposing a diffusion barrier that impedes ATA access to the substrates. Despite conducting simultaneous oxidation and transamination reactions herein to reduce the acetal bond formation, evidence of gel formation was observed (Additional file 1: Fig. S5A). In an effort to further reduce gel formation that consumes oxidized intermediates through side reactions, a series of experiments were performed to improve the balance of FgrGaOx and SpATA used in the reaction. Measured using the 2,2′-azino-bis(3-ethylbenzothiazoline-6-sulfonic acid) assay (the ABTS assay), the activity of FgrGaOx on galactose was 69,100 ± 2100 U/g with no significant inhibition by the aminated product, which is equivalent to 2.06 ± 0.06 U/mL under our experimental setup. In comparison, units of SpATA activity on oxidized galactose was 58 ± 2 U/g, equivalent to 0.009 ± 0.0002 U/mL in the cascade reaction or less than 0.5% of FgrGaOx activity.

**Fig. 7:**
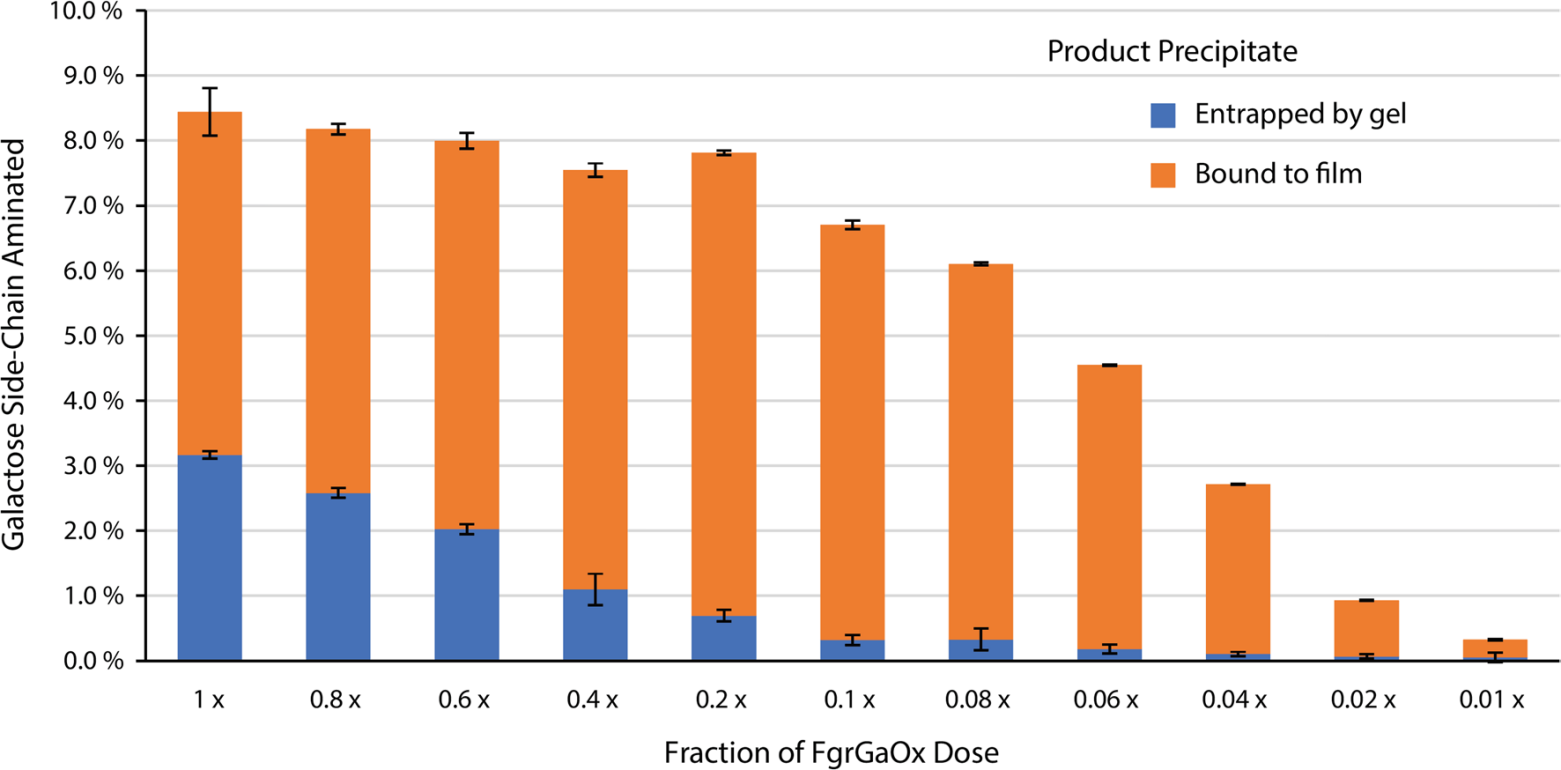
Conversions of galactomannan amination catalyzed by FgrGaOx with reduced doses and SpATA measured using the Q-NPEA assay. Precipitate formation were measured separately from the transparent adhesive film and the liquid suspension. Reactions (200 µL) comprised 50 mM HEPES buffer (pH 7.5), 20 µM PLP, 10 mM NPEA, 0.15 mg/mL SpATA, 0.29% galactomannan equivalent to 5 mM galactose and FgrGaOx at various doses. The 1.0 × FgrGaOx dose corresponds to 29.8 µg/mL FgrGaOx, 12.8 µg/mL catalase, and 1.8 µg/mL HRP. All reactions were carried out at 37 °C and 600 rpm on 96-well plates (*n* = 4, error bars indicate standard errors)

**Fig. 8:**
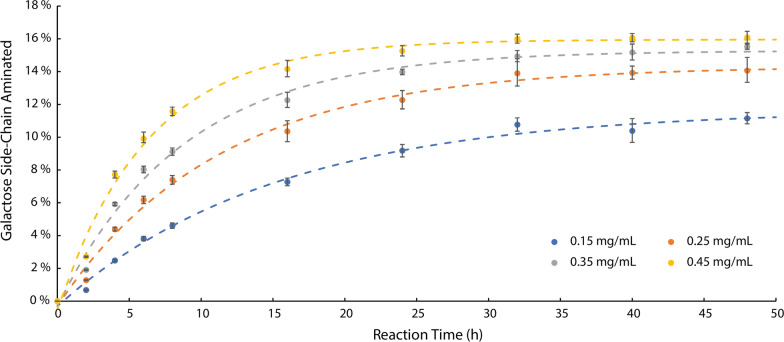
Conversions of galactomannan amination catalyzed by FgrGaOx and SpATA with increasing SpATA dose measured using the Q-NPEA assay. The dotted lines represent the best-fitted exponential functions. Reactions (200 µL) comprised 50 mM HEPES buffer (pH 7.5), 20 µM PLP, 10 mM NPEA, 29.8 µg/mL FgrGaOx, 12.8 µg/mL catalase, 1.8 µg/mL HRP, 0.29% galactomannan which contains 5 mM galactose and SpATA at concentrations shown in the figure legend. All reactions were carried out at 37 °C and 600 rpm on 96-well plates (*n* = 4, error bars indicate standard errors)

The impact of decreasing FgrGaOx loading and increasing SpATA loading on galactomannan amination was studied using the Q-NPEA assay (Fig. 7). To track the reduction in gel formation, product precipitate formed on the transparent adhesive film and in the suspension (i.e., trapped in the gel formed after oxidation) were quantified by separately measuring the optical absorbance of the adhesive film and the assay solution. Reducing the FgrGaOx loading by 80% reduced gel formation by 76.4%. The product formation, on the other hand, dropped by only 7.5%. Compared to the original FgrGaOx dose, aminated galactomannan produced using 20% of the original FgrGaOx loading was observably more homogeneous and fluid-like (Additional file 1: Fig. S5B). Further reducing the FgrGaOx dose resulted in a linear reduction in product formation. Increasing the SpATA dose resulted in a linear increase in product formation over 4 h (Fig. 8) and increasing the SpATA dose three times (from 0.15 to 0.45 mg/mL) increased the product yield after 32 h by 44%. Notably, for all tested ATA loadings, reactions stopped after 32 h regardless of the aminated galactomannan yield or the amount of remaining oxidized substrate.

### Mutant design and testing

**Fig. 9:**
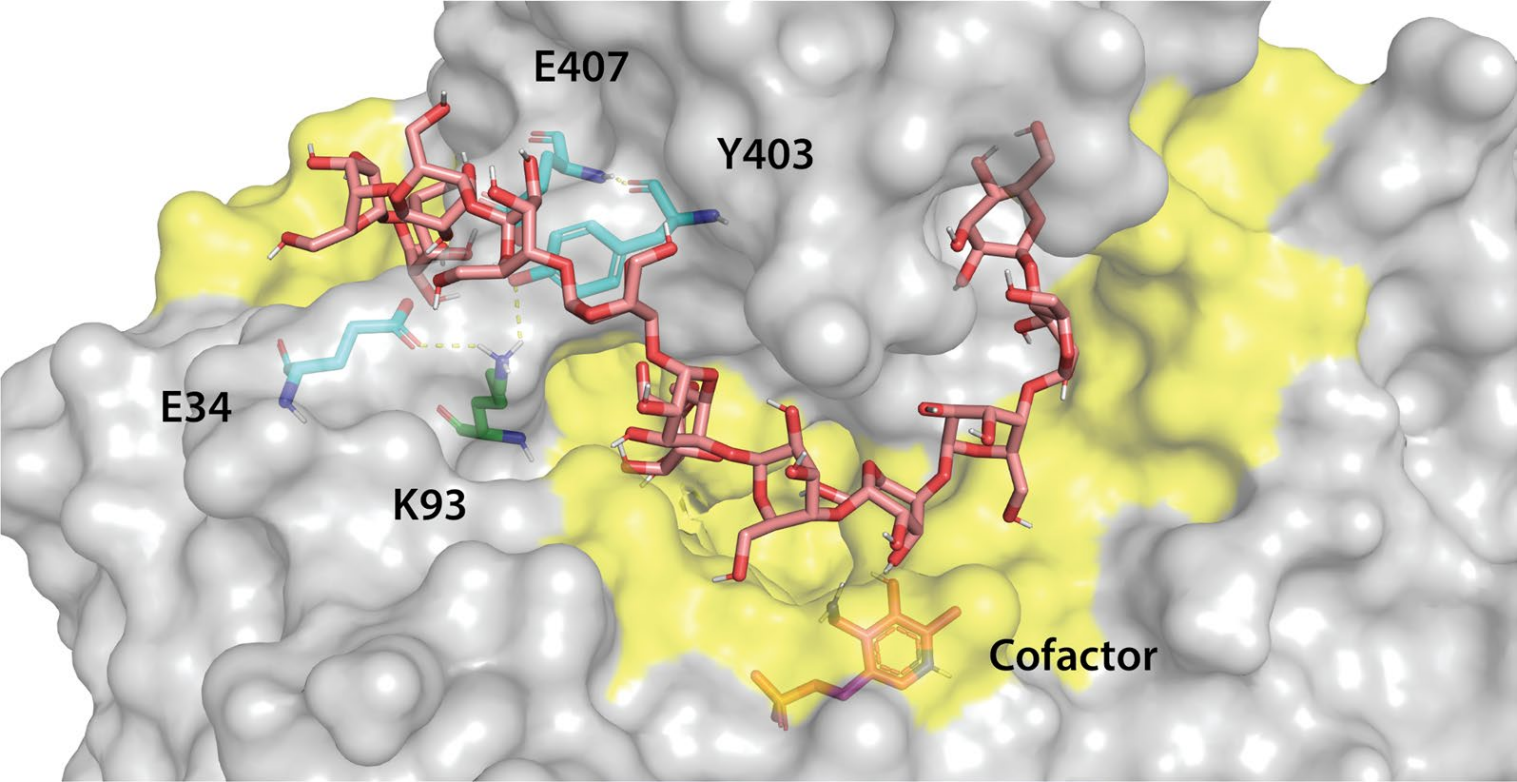
Four hydrophilic amino acids on the SpATA surface blocking the binding of galactomannan oligosaccharide to the predicted polysaccharide binding cleft. The polysaccharide binding cleft is indicated by the yellow shade on the enzyme surface. Amino acids in green and cyan are from different polypeptide chains. The SpATA structure was obtained from the RCSB PDB database (PDB ID: 3HMU) and the sulfate ions in active sites were replaced with PMP to construct the holoenzyme. A galactomannan oligosaccharide with an 11-mannose backbone and one galactose side chain on the fifth mannose unit connected via α-(1→6)-linkage was used in the docking simulation

Given the higher yield of SpATA-catalyzed aminations compared to CvATA, SpATA serves as a preferred starting point for protein engineering aimed at enhancing the enzymatic synthesis of aminated polysaccharides from plant sources. Inspection of the SpATA surface revealed closely positioned hydrophobic and aromatic residues near the active site entrance that could form a potential polysaccharide binding cleft. Using docking simulations, four hydrophilic amino acids were identified as blocking polysaccharide access to the putative binding surface (Additional file 1: Fig. S6 and Fig. 9). To maintain the hydrophobicity of the region and reduce the steric hindrance of polysaccharide binding, four alanine substitutions (E34A, K93A, Y403A and E407A) were created following an alanine scanning strategy [28].

**Fig. 10:**
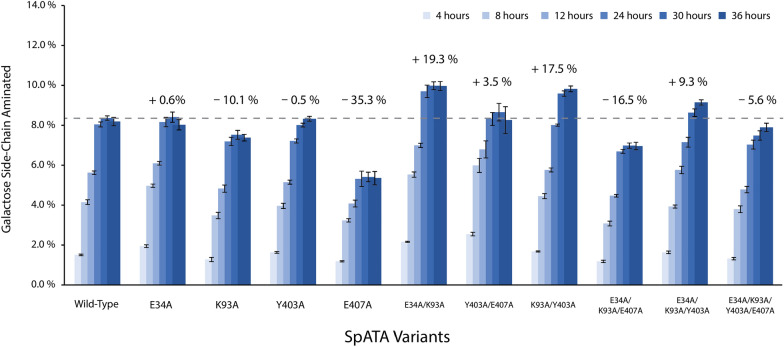
Conversions of galactomannan amination catalyzed by FgrGaOx and SpATA mutants measured using the Q-NPEA assay. The dashed line indicates the conversion achieved by the wild-type SpATA in 30 h, and percentages indicate how 30-h conversions achieved by SpATA variants compare to the wild-type SpATA. Reactions (200 µL) consisted of 50 mM HEPES buffer (pH 7.5), 29.8 µg/mL FgrGaOx, 12.8 µg/mL catalase, 1.8 µg/mL HRP, 0.15 mg/mL ATA, 10 mM NPEA, 20 µM PLP and 0.29% galactomannan containing 5 mM galactose. Reactions were conducted at 37 °C and 600 rpm on a 96-well plate (*n* = 4, error bars indicate standard errors)

Ten SpATA mutants were constructed, including four single mutants (E34A, K93A, Y403A, E407A), three double mutants (E34A/K93A, K93A/Y403A, Y403A/E407A), two triple mutants (E34A/K93A/Y403A, E34A/K93A/E407A), and one mutant containing all four amino acid substitutions. The Q-NPEA assay was used to quantify the formation of aminated products from FgrGaOx-oxidized galactomannan (Fig. 10). Compared to wild-type SpATA, single mutations E34A, K93A and Y403A showed no or detrimental effects on product yield, whereas mutants with combined mutations E34A/K93A, K93A/Y403A and E34A/K93A/Y403A increased product yield after 30 h by 9.3–19.3%. Most notably, the E407A substitution reduced product yield of wild-type SpATA by 35.3%; moreover, introducing the E407A substitution to E34A/K93A and E34A/K93A/Y403A reduced corresponding product yields by 29.0% and 13.7%, respectively.

## Discussion

Amine transaminases are versatile biocatalysts that have the potential to substitute harmful chemical catalysts and harsh reaction conditions in the production of high-value chiral amines. The substrate scope of ATAs is typically evaluated using the acetophenone assay [29], a well-established method relying on the detection of UV-absorbing acetophenone when the amino donor 1-phenylethylamine (1-PEA) is deaminated. Although the assay is highly sensitive and easy to deploy, acetophenone evaporation and limitations on enzyme concentration and UV-absorbance of the amino acceptor are major drawbacks affecting the versatility of the assay [30]. The NPEA assay developed by Baud et al. [27] offers an alternative way of ATA screening. By generating a colored precipitate, the assay provides visual confirmation of reaction progress, however, the formation of an opaque suspension means that it can only be used for semi-quantitative comparisons of ATA activity [30]. In this study, the Q-NPEA assay was established, which builds from the NPEA assay and enables accurate quantification of the reaction product. Compared to other colorimetric assays, such as the Ehrlich test presented by Cairns et al. [30] which requires the addition of the Ehrlich reagent containing concentrated HCl and alcohol to measure indole formation, the Q-NPEA assay is easy to set up and allows continuous conversion measurements without terminating the reaction using additional reagents. Additionally, the selective binding of product precipitates to the transparent adhesive film allows for ATA screening on insoluble substrates, such as oxidized cellulose and other biopolymers, which are often difficult compounds to screen using spectrophotometric assays.

Using the Q-NPEA assay, the activity of both CvATA and SpATA towards oxidized polysaccharides was confirmed, specifically oxidized galactomannan and xyloglucan, on which activities were comparable to those measured on oxidized oligosaccharides. Although CvATA showed higher initial activity, SpATA remained active over longer reaction times, which led to higher product yield and a higher degree of polysaccharide amination. By balancing the FgrGaOx and SpATA loadings, gel formation in simultaneous galactomannan oxidation–transamination reactions was significantly suppressed without negatively impacting the product yield. Simply increasing SpATA concentration increased product yield; however, enzyme deactivation after 32 h regardless of SpATA loading suggests that low operational stability is the main factor limiting the enzyme performance (Fig. 8).

**Fig. 11:**
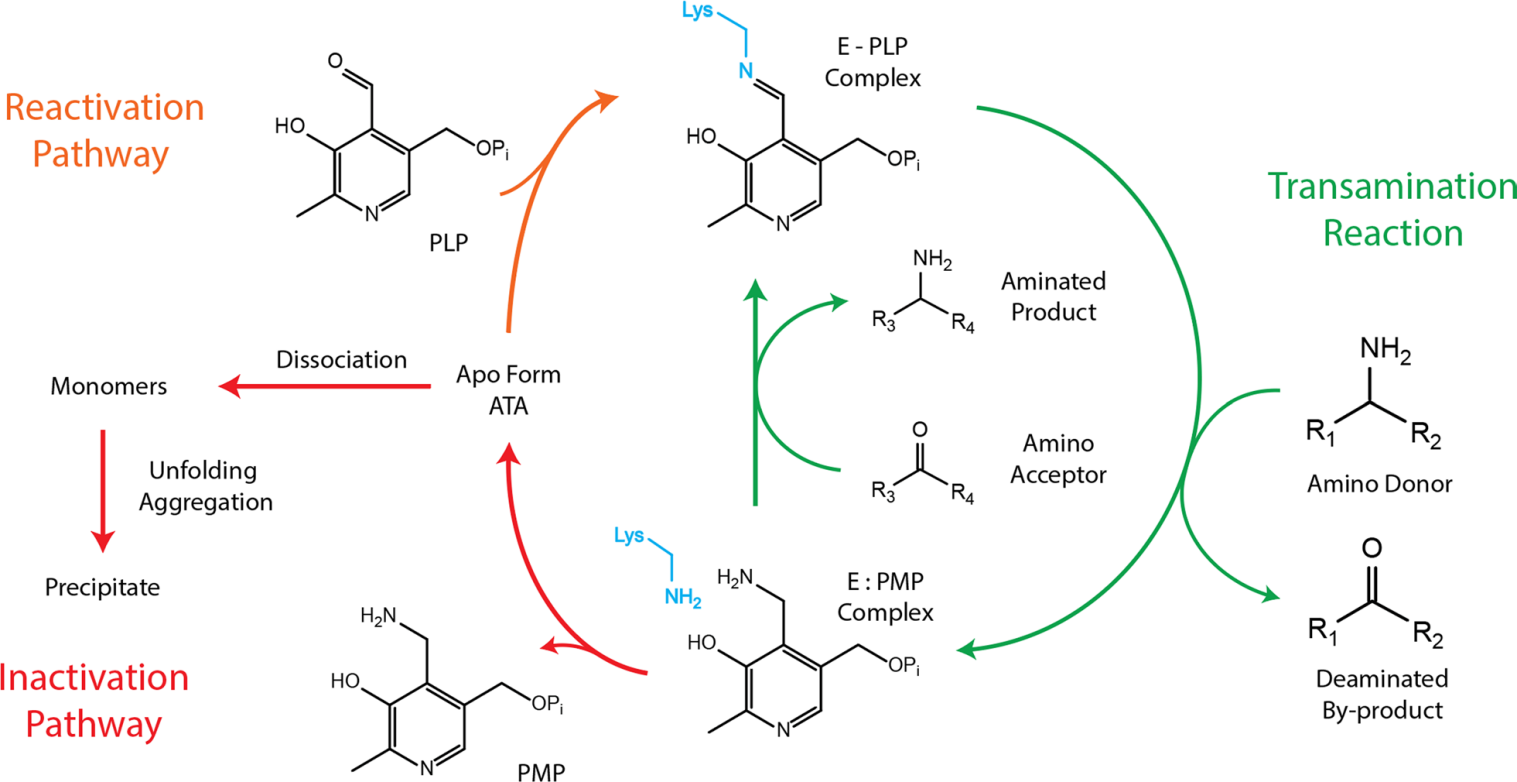
The postulated ATA deactivation mechanism reproduced from Börner et al. [31]

CvATA deactivation was previously investigated and is believed to result from dimer dissociation [31, 32]. The “phosphate group binding cup” (PGBC) in the ATA active site consists of conserved amino acids that bind the PLP/PMP cofactor and maintain the integrity of the ATA structure via non-covalent interactions bridged by the bound cofactors (Additional file 1: Fig. S7) [33–35]. According to Börner et al. [31], the formation of the less stable ATA–PMP complex in the first half-transamination reaction allows PMP to escape, which destabilizes the PGBC and can lead to the dissociation, unfolding, and irreversible deactivation of the ATA (Fig. 11). This mechanism explains CvATA deactivation and the discoloration of the reaction media (Additional file 1: Fig. S8) with PLP depletion, which is supported by the ESI-Q-MS detection of PMP-derived compounds in discolored reaction medium (Additional file 1: Fig. S9) [31]. For SpATA, however, PMP formation was not detected in reactions containing only (*S*)-1-PEA and the PLP cofactor, suggesting an alternative deactivation pathway that involves the amino acceptor. Evidently, the creation of SpATA variants with improved operational stability will require a deeper understanding of the ATA inactivation mechanism.

To date, attempts to engineer ATAs have primarily focused on enhancing the catalytic efficiency and broadening the substrate scope of ATA for better acceptance of monomeric substrates with bulky substituents [36–41]. Strategies have focused on engineering the substrate binding pockets, whereas positions distant from the active site are often overlooked. Herein, a structure-based approach was used to modify surface-exposed amino acids on SpATA that could improve the binding of polysaccharide substrates to the enzyme surface. Although single mutations did not show positive effects on enzyme activity, mutants with multiple alanine substitutions other than the E407A increased the yield of aminated galactomannan. Saturation mutagenesis could be used at the positions identified herein to further improve SpATA activity on oxidized polysaccharides. Notably, the *k*_cat_ of the E407A variant (1.9 ± 0.2 s^−1^) on pyruvate was 2.5 times lower than wild-type SpATA (4.8 ± 0.7 s^−1^) (Additional file 1: Fig. S10), which may be attributed to the location of E407A mutation near the interfacial loops that stabilize the dimeric structure of SpATA (Additional file 1: Fig. S11) [42].

Although the SpATA activity on oxidized carbohydrates may be improved through further enhancing substrate binding, the low operational stability and low affinity of the enzyme towards the PLP and PMP cofactors remain problems affecting its applications in industrial processes. In general, amino acid sequences of fold type I ATAs are highly conserved at the cofactor binding pocket near the PGBP [33–35] but are less conserved near the pyridinium ring binding motif. A study by Roura et al. [43] identified a single point mutation (V124N) in CvATA that enhances hydrogen bonding with the PMP pyridinium nitrogen, increasing the enzyme’s affinity to PMP by 3 times. The equivalent position in SpATA is already an asparagine, which may explain the comparatively high operational stability of SpATA. Nevertheless, maximizing SpATA catalytic cycles and product yield from the oxidation–transamination cascade required an exogenous supply of PLP, justifying enzyme engineering efforts that focus on further increasing the cofactor affinity of SpATA.

## Conclusions

Using a one-pot oxidation–transamination cascade, this work established a fully enzymatic pathway for aminations of plant-derived polysaccharides. Validated using HPLC-RI and a GC–MS-based deuterium labeling method, the Q-NPEA assay developed herein enabled simple and accurate time-course yield measurements for transamination reactions. Balancing the activity of FgrGaOx and ATAs used in the cascade effectively suppressed gel formation without substantially reducing product yield. Via rational design, alanine substitutions were performed on selected surface amino acids of SpATA which have potential effects on polysaccharide binding. Mutants with multiple alanine substitutions other than E407A produced up to 20% higher product yield. The E407A mutation showed detrimental effects on SpATA activity, and the position may have important functions in maintaining the dimeric enzyme structure. The low operational stability of ATAs, caused by low affinity towards the PLP and PMP cofactors, was identified as a major problem limiting the yield of aminated products. To increase the applicability of the designed cascade in industrial settings, amino acid positions in ATA structure affecting the cofactor binding need to be identified and exploited.

## Methods

### Materials

Galactomannan (P-GGM28) and xyloglucan (P-XYGLN) were purchased from Megazyme (Ireland); Catalase (60635), Horse Radish Peroxidase (HRP) (P6782), pyruvate and carbohydrate substrates were purchased from Sigma-Aldrich (Germany). All buffering chemicals and growth medium were obtained from BioShop (Canada). Complementary primers used for site-directed mutagenesis were ordered from IDT (USA). The QuikChange mutagenesis kit and Miniprep plasmid extraction kit were obtained from Agilent (US) and Qiagen (Netherlands), respectively. The electrocompetent and chemically competent *Escherichia coli* cells were produced in-house. The FgrGaOx was produced as previously reported [44] and kindly provided by O. Mototsune (University of Toronto). All other chemicals were purchased from Sigma-Aldrich (Germany).

### Production and purification of amine transaminases from *Chromobacterium violaceum* (CvATA) and *Silicibacter pomeroyi*  (SpATA)

The ATA production and purification protocol was adopted from Aumala et al. [13] with minor modifications. Briefly, the *E*. *coli* BL21(DE3) strains containing pET-29(a)+ and pET-22(b)+ plasmids subcloned with codon-optimized CvATA and SpATA genes, respectively, were cultivated in LB media (Miller) supplemented with 50 µg/mL kanamycin and 100 µg/mL ampicillin, respectively. The cultures were grown at 37 °C until the optical density at 600 nm reached 0.6–0.8, and enzyme productions were induced by adding 1 mM isopropyl-β-d-thiogalactopyranoside (IPTG) to the cell cultures. The inductions were conducted for 15–16 h at 30 °C and 220 rpm. The cells were pelleted by centrifugation, resuspended in the lysis buffer (50 mM potassium phosphate at pH 7.5, supplemented with 0.1 mM PLP and 20 mM imidazole), and sonicated to release the intracellularly expressed ATAs.

After cell lysis, the lysates were clarified by centrifugation (15,000×*g*, 90 min), and the ATAs were purified using Ni–NTA columns (the washing step was done with the lysis buffer, and the elution buffer contains 50 mM potassium phosphate at pH 7.5, supplemented with 0.1 mM PLP and 500 mM imidazole). Buffer exchange was performed using 50 mL centrifuge filters with 10 kDa MWCO, and the purified ATAs were stored in 50 mM potassium phosphate (pH 7.5) buffer containing 0.1 mM PLP. The concentration and purity of the purified ATAs were determined using the Bradford assay and SDS-PAGE, respectively. The ATAs were diluted to 30 mg/mL, aliquoted, and stored at − 80 °C until further use.

### Activity measurements via the acetophenone assay

The activity of ATAs towards selected amino acceptors was measured using the acetophenone assay, which measures the rate of acetophenone generation by measuring the UV-absorbance of the assay mixture at 245 nm. Assay mixtures contained 50 mM HEPES buffer (pH 7.5), 5 mM (*S*)-1-PEA, 20 µM PLP, and 5 mM pyruvate or selected carbohydrates containing 5 mM galactose. When oxidized carbohydrates were used as the amino acceptor, 29.8 µg/mL FgrGaOx, 12.8 µg/mL catalase, and 1.8 µg/mL HRP were also added. The assay mixtures were pre-oxidized at 37 °C for an hour, and transamination reactions were carried out on a UV-compatible 96-well microtiter plate at 200 µL volumes. For each reaction, 30 µg ATA was added (300 ng when using pyruvate as the amino acceptor), and the plate was moved immediately to a plate reader operating at 37 °C without shaking. The absorbance readings were taken at 245 nm in 30-s intervals for 30 min, and linear portions of the absorbance-vs-time graphs were used to calculate the rates of acetophenone formation. All experiments were conducted in 4 replicates (*n* = 4).

### ATA kinetic parameter measurements via the acetophenone assay

The kinetic parameters of ATAs were measured using the acetophenone assay and using pyruvate with concentrations from 0.001 to 0.1 mM as the amino acceptor. The reactions were performed in the same way as in the activity measurements except for using variable pyruvate concentration. Reactions (200 µL) were performed on a UV-compatible 96-well microtiter plate at 37 °C without shaking. The UV-absorbance of the assay mixtures was measured at 245 nm in 20-s intervals for 10 min, and the initial linear portion of absorbance-vs-time graphs was used to calculate the rate of acetophenone formation. The initial rates were plotted against pyruvate concentrations, and kinetic parameters were calculated using Prism 10 software.

### Conversion measurements via the Q-NPEA assay

The conversions of transamination reactions were measured using the Q-NPEA assay. The assay mixtures were prepared the same way as in the acetophenone assay except that 10 mM NPEA was used instead of 5 mM (*S*)-1-PEA. The enzymes, including FgrGaOx, catalase, HRP, and ATAs were added at variable loadings depending on the objective of the experiment. The assay reactions were performed in 200 µL volumes in a transparent 96-well microtiter plate. After loading all assay components, the plate was sealed using a piece of transparent and adhesive film (Bio-Rad MSB-1001) and was incubated with the film side facing down in a thermomixer at 37 °C and 600 rpm. The absorbance of reactions was measured at 440 nm using a plate reader at 9 different points with the film side facing up, and the absorbance values were averaged and converted to NPEA consumption using a standard curve (described below). Normally, absorbance readings are taken with the film attached to the plate. For the experiment described in Fig. 7, however, the film was peeled off, rinsed with water, and re-applied onto a clean plate, and absorbance readings were taken separately from both plates.

The standard curve was constructed using 0.1 to 1.0 mM pyruvate as the amino acceptor and 10 mM NPEA as the amino donor. Besides pyruvate and NPEA, assay mixtures also contained 50 mM HEPES, 20 µM PLP, and 0.15 mg/mL SpATA. The reactions were performed in 200 µL volumes following the Q-NPEA assay protocol. The absorbance of each reaction was measured at 440 nm and mapped to the corresponding initial pyruvate concentration. The assay mixtures were analyzed in parallel using HPLC-RI, and complete pyruvate depletion was confirmed for reactions with all pyruvate concentrations. All experiments were performed with 4 replicates (*n* = 4).

### Confirmation of galactose aminations by ESI-Q-TOF MS

Aminated galactose samples for ESI-Q-TOF MS analysis were produced in 1 mL volumes using (*S*)-1-PEA as the amino donor. The reaction mixtures were prepared the same way as in the acetophenone assay. The reactions were performed in 1.5 mL microcentrifuge tubes on a thermomixer operating at 37 °C and 600 rpm for 5 h. The products were purified by filtration using a Pall Nanosep centrifuge filter (1 kDa cutoff) and diluted 10 times prior to the MS analyses (the diluted samples contained 5 mM HEPES buffer and 0.5 mM original/oxidized/aminated galactose). The ESI-Q-TOF MS data were acquired by the BioZone mass spectrometry facility (University of Toronto) using a Q Exactive Orbitrap mass spectrometer (Thermo Scientific) with nano-electrospray ionizer in positive mode. Data analysis and visualization were performed using the Xcalibur software. All samples were produced in triplicates (*n* = 3).

### Confirmation of galactomannan amination by X-ray photoelectron spectroscopy

Aminations of galactomannan were performed the same way as galactose aminations except for using 50 mM potassium phosphate buffer (pH 7.5) instead of HEPES and a longer reaction time of 24 h. The oxidized reference was produced without the addition of ATA and the non-oxidized reference was produced without any enzyme. After reactions, SpATA was added to the oxidized reference, and SpATA, FgrGaOx, catalase, and HRP were added to the non-oxidized reference to make the references have the same composition as aminated samples. The products were desalted using Amicon Ultra-0.5 centrifuge filters (100 kDa cutoff) to remove proteins and residual amino donors and were washed repetitively until the calculated buffer concentration fell below 0.1% of the original value (1000× dilution). The desalted products were freeze–dried, and the XPS analyses were performed by OCCAM (University of Toronto) using a K-Alpha X-ray photoelectron spectrometer system (Thermo Scientific).

### Galactose oxidase activity measurement using the ABTS assay

The ABTS assay was conducted in 250 µL volumes on a transparent 96-well microtiter plate. The assay mixture comprised 50 mM HEPES buffer (pH 7.5), 5 mM galactose, 2 mM ABTS, 1.8 µg/mL HRP, and 29.8 ng/mL FgrGaOx. Before adding galactose, the mixtures were incubated at 30 °C for 15 min to activate galactose oxidase. After adding all components, the microtiter plate was immediately transferred to a plate reader operating at 30 °C, where the absorbance of reactions was measured at 420 nm for 30 min in 30-s intervals without shaking. The activity of FgrGaOx was calculated from the linear portion of the absorbance-vs-time graph. In the product inhibition experiment (Additional file 1: Fig. S12), 5 mM C-2 glucosamine was added to the assay reaction as a model aminated product. All other components and procedures remain the same.

### Degree of galactomannan amination measurement using a GC–MS-based deuterium labeling method

The degree of galactomannan amination was measured by comparing the DO prior to and after transamination reactions. The DO of galactomannan was measured using a GC–MS-based deuterium labeling method described in Parikka et al. [15], and the protocol described in the paper was adopted with minor changes. Briefly, oxidation and transamination reactions were performed in series using the same reaction mixture and experimental setup as used in XPS sample preparations. During the oxidation stage, 250 µL reaction mixtures containing 59.6 µg/mL FgrGaOx, 25.6 µg/mL catalase, 3.6 µg/mL HRP, and 0.58% galactomannan (equivalent to 10 mM galactose) was transferred to a 2-mL centrifuge tube and was incubated at 37 °C and 600 rpm for 4 h in a thermomixer without controlling the oxygen level. The product was heated to 100 °C for 5 min to deactivate the oxidase, and other reaction components were added. The final composition of the reaction (500 µL) was the same as that used for preparing XPS samples. After adding the ATA, the reaction was again incubated at 37 °C and 600 rpm for 12 h. Following the transamination reaction, 3 mg of NaBD_4_ was added to each reaction to reduce the remaining oxidized galactose back to the non-oxidized form and have a deuterium atom replacing a hydrogen atom in the C-6 hydroxyl group. The reactions were then stirred overnight at room temperature. After the reduction, the remaining NaBD_4_ in the product was neutralized by adding 15 µL acetic acid. Galactomannan was precipitated using 70% ethanol and collected by centrifugation. The precipitated galactomannan was dried under a stream of nitrogen at 40 °C and acid methanolyzed using 2 mL methanolysis reagent at 100 °C for 3 h. The degraded product was again dried under a stream of nitrogen at 40 °C, and silylation was performed by adding 1 µL trimethylsilyl chloride, 99 µL *N*,*O*-Bis(trimethylsilyl)trifluoroacetamide, and 100 µL pyridine to each sample and incubating the samples for 1 h at 60 °C. The silylated product was dried under nitrogen at room temperature and dissolved in 1 mL of heptane. The final products were filtered using 0.22 µm filters and analyzed using GC–MS.

The GC–MS (Agilent 5977B MSD no FID, DB-5HT column) was performed at 17 psi using 150 °C helium as the elution gas. The column temperature profile was set to 150 °C (3 min)–2 °C/min–180 °C–4 °C/min–200 °C (5 min). Under this setup, the galactose peak appeared 11.6 min after elution started, with *m*/*z* 361 and *m*/*z* 362 peaks corresponding to the non-oxidized and oxidized galactose, respectively. Using the benchmarking data from Parikka et al. [15], the DO of galactomannan was calculated as:

1 $$\begin{array}{*{20}c} {{\text{Oxidation}}\;\% = \frac{B - A/3}{{B + 2A/3}} \times 100 \% } \\ \end{array} ,$$

where *A* and *B* are the relative abundance of the *m*/*z* 361 and *m*/*z* 362 peaks, respectively. For aminated galactomannan, the DO needs to be corrected for amination using:

2 $$\begin{array}{*{20}c} {{\text{Oxidation }}_{{{\text{Corr}}}} \;\% = {\text{Oxidation}}\;\% \times \frac{{1 - {\text{Oxidation}}_{{ {\text{ref}}}} \;\% }}{{1 - {\text{Oxidation }}\;\% }}} \\ \end{array} ,$$

where oxidation percentage ${\text{Oxidation \% }}$ is the value calculated using Eq. (1), and reference oxidation percentage $\left( {{\text{Oxidation}}_{{ {\text{ref}}}} \;{\text{\% }}} \right)$ is the DO measured for the oxidized reference (with no ATA). The degree of galactomannan amination was then calculated as the differences between the corrected and reference oxidation percentages.

### Identification of amino acids important for polysaccharide binding using docking simulation

The substrate docking simulations were performed using AutoDock FR [45]. The apo-form SpATA structure was obtained from the RCSB PDB database and modified by substituting the sulfate ions in active sites with PLP or PMP to generate holo-form structures. The SpATA mutants were created using the mutagenesis function and energy-minimized using the optimize function in Pymol. The receptor files were prepared by removing water and non-polar hydrogens, and the locations of binding pockets were determined using the AutoDock Tools. A galactomannan oligosaccharide with an 11-mannose backbone and one galactose connected to the fifth mannose unit via α-(1→6)-linkage was used in the docking simulation. The simulations were performed with an exhaustiveness of 16 and the results were visualized in Pymol.

### Site-directed mutagenesis and transformation into *E. coli* hosts

The genes encoding the SpATA mutants were produced using the Agilent QuikChange site-directed mutagenesis kit and following the manufacturer’s protocol with minor modifications. A list of primers and their properties, as well as the PCR reaction settings, are summarized in Additional file 1: Table S1. Using the Qiagen Miniprep DNA extraction kit, the template plasmids were extracted from the corresponding *E. coli* DH5α strains, and the concentrations of the templates were measured using a nanodrop spectrophotometer (Thermo Scientific). The PCR reactions were performed following the manufacturer’s protocol, and products were analyzed using DNA agarose gel to confirm successful gene amplification. The amplified products were digested with DpnI endonuclease to remove the templates and transformed into electrocompetent *E. coli* DH5α cells via electroporation. The transformed strains were cultivated in SOC medium for 1 h and inoculated on LB (Miller) plates supplemented with  0.15 mg/mL ampicillin. Successfully transformed hosts were further cultivated in liquid media, and their plasmids were extracted and sequenced to verify the incorporation of designed mutations. Plasmids with designed mutations were then transformed into chemically competent *E. coli* BL21(DE3) cells, which were again cultivated in SOC medium and plated on LB plates with 0.15 mg/mL ampicillin and stored in 25% (v/v) glycerol at − 80 °C until further use.

### Supplementary Information

**Additional file 1: Figure S1.** Acetophenone evaporation at 37 °C. **Figure S2.** The initial activity of CvATA and SpATA. **Figure S3.** Absorption spectra of red precipitates formed in the Q-NPEA assay. **Figure S4.** HPLC results confirming pyruvate depletion. **Figure S5.** Gel formation by acetyl bond formation in oxidized intermediates. **Figure S6.** Docking of galactomannan oligosaccharide on SpATA surface before and after alanine substitutions. **Figure S7.** PGBC in SpATA hosting the PLP cofactor. **Figure S8.** Discoloration of PLP catalyzed by CvATA. **Figure S9.** Formation of PMP-derived compounds in the discolored PLP solution. **Figure S10.** Initial rates of the wild-type SpATA and SpATA E407A measured on pyruvate with varying concentrations. **Figure S11.** The location of E407A mutation near the interfacial loops. **Figure S12.** Galactose oxidase inhibition by the aminated product measured using the ABTS assay. **Table S1.** A list of primers used in the site-directed mutagenesis of SpATA.

## Data Availability

The datasets generated for this study are available on request to the corresponding author.

## Abbreviations

ABTS: 2,2′-Azino-bis(3-ethylbenzothiazoline-6-sulfonic acid)
ATA: Amine transaminase
ESI-Q-TOF MS: Electrospray-ionization quadrupole time-of-flight mass spectrometry
GaOx: Galactose oxidase
GC–MS: Gas chromatography–mass spectrometry
HPLC-RI: High-performance liquid chromatography refractive index
HRP: Horseradish peroxidase
NPEA: 2-(4-Nitrophenyl)ethan-1-amine
PCR: Polymerase chain reaction
PEA: Phenylethylamine
PGBC: Phosphate group binding cup
PLP: Pyridoxal 5-phosphate
PMP: Pyridoxamine 5-phosphate
XPS: X-ray photoelectron spectroscopy
