# Supplementary material for: Biocatalytic cascade to polysaccharide amination
Source: Biotechnol Biofuels Bioprod. 2024 Feb 27;17:34. doi: 10.1186/s13068-024-02477-6 (PMC10898118; doi:10.1186/s13068-024-02477-6)
Supplement: Supplementary file 1 — Additional file 1: Figure S1. Acetophenone evaporation at 37 °C. Figure S2. The initial activity of CvATA and SpATA. Figure S3. Absorption spectra of red precipitates formed in the Q-NPEA assay. Figure S4. HPLC results confirming pyruvate depletion. Figure S5. Gel formation by acetyl bond formation in oxidized intermediates. Figure S6. Docking of galactomannan oligosaccharide on SpATA surface before and after alanine substitutions. Figure S7. PGBC in SpATA hosting the PLP cofactor. Figure S8. Discoloration of PLP catalyzed by CvATA. Figure S9. Formation of PMP-derived compounds in the discolored PLP solution. Figure S10. Initial rates of the wild-type SpATA and SpATA E407A measured on pyruvate with varying concentrations. Figure S11. The location of E407A mutation near the interfacial loops. Figure S12. Galactose oxidase inhibition by the aminated product measured using the ABTS assay. Table S1. A list of primers used in the site-directed mutagenesis of SpATA. [file 13068_2024_2477_MOESM1_ESM.docx]

Biocatalytic Cascade to Polysaccharide Amination

Additional File 1: Supplementary Figures and Tables

**Figure S1.** Acetophenone Evaporation at 37 °C. Acetophenone solutions (200 µL) with concentrations from 0.1 to 0.5 mM were prepared and transferred to a UV-compatible microtiter plate. The plate was moved to a plate reader operating at 37 °C and incubated without shaking. The absorbance of solutions was monitored continuously at 245 nm for 3 h in 5 min intervals. The readings were converted to acetophenone concentrations using a standard curve.


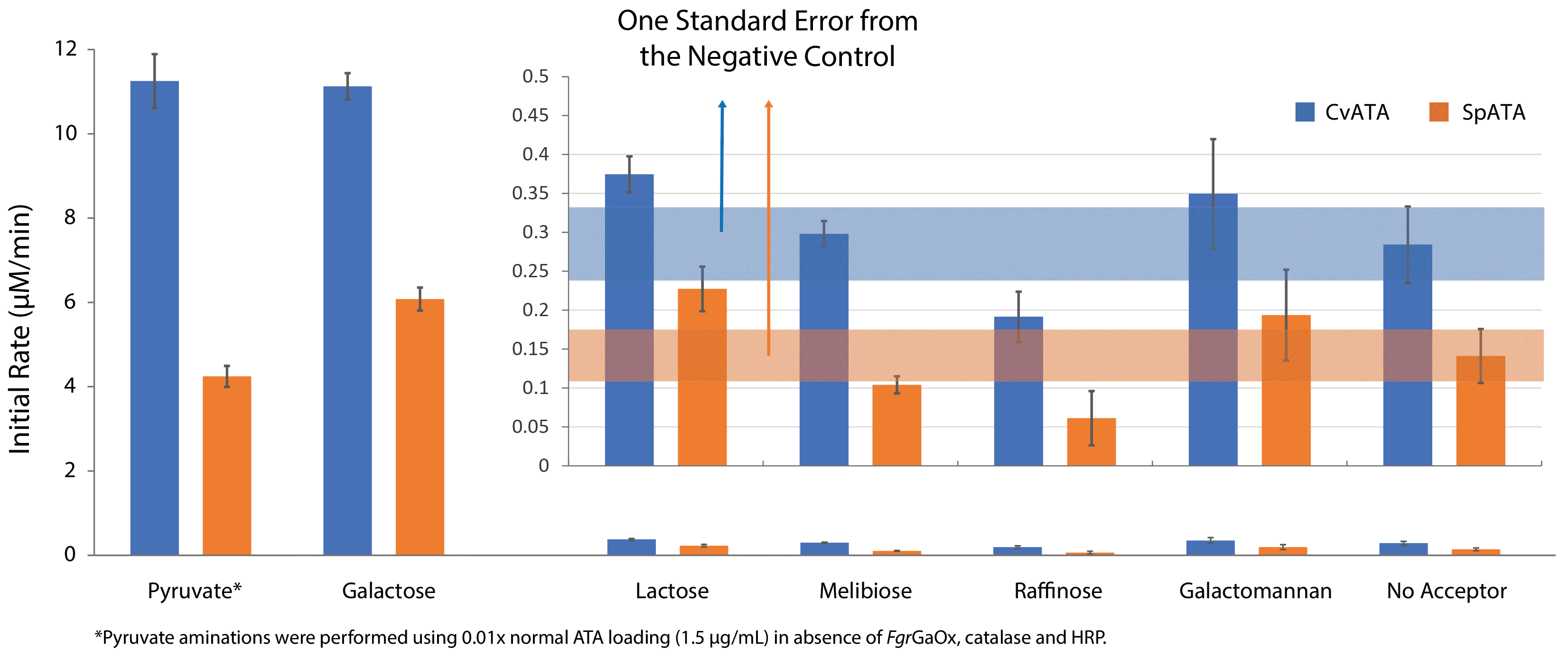


**Figure S2.** Initial activity of CvATA and SpATA measured using the acetophenone assay with pyruvate and FgrGaOx oxidized carbohydrates as amino acceptors. The “No Acceptor” groups refer to negative controls that lack the amino acceptor, and from which an increasing background was observed at 245 nm. Reactions were conducted the same way as in the acetophenone assay described in the method section. Error bars indicate standard errors, and shaded areas are within one standard error from the increasing background. As shown in the inserted graph, initial rate of neither ATA is significant on oxidized oligo- and polysaccharides. All experiments were conducted with 4 replicates (n = 4).

**Figure S3.** Absorption spectra of red precipitate layers generated in the Q-NPEA assay. The peak absorbance lies between 410 and 440 nm depending on the ATA being screened. Since CvATA has lower operational stability, SpATA was used to construct the standard curve, and absorbance at 440 nm was used to measure the yield of all reactions. Reactions were performed following the standard Q-NPEA assay protocol for 24 h, and absorbance of reaction wells was measured from 380 to 480 nm in 5 nm steps.


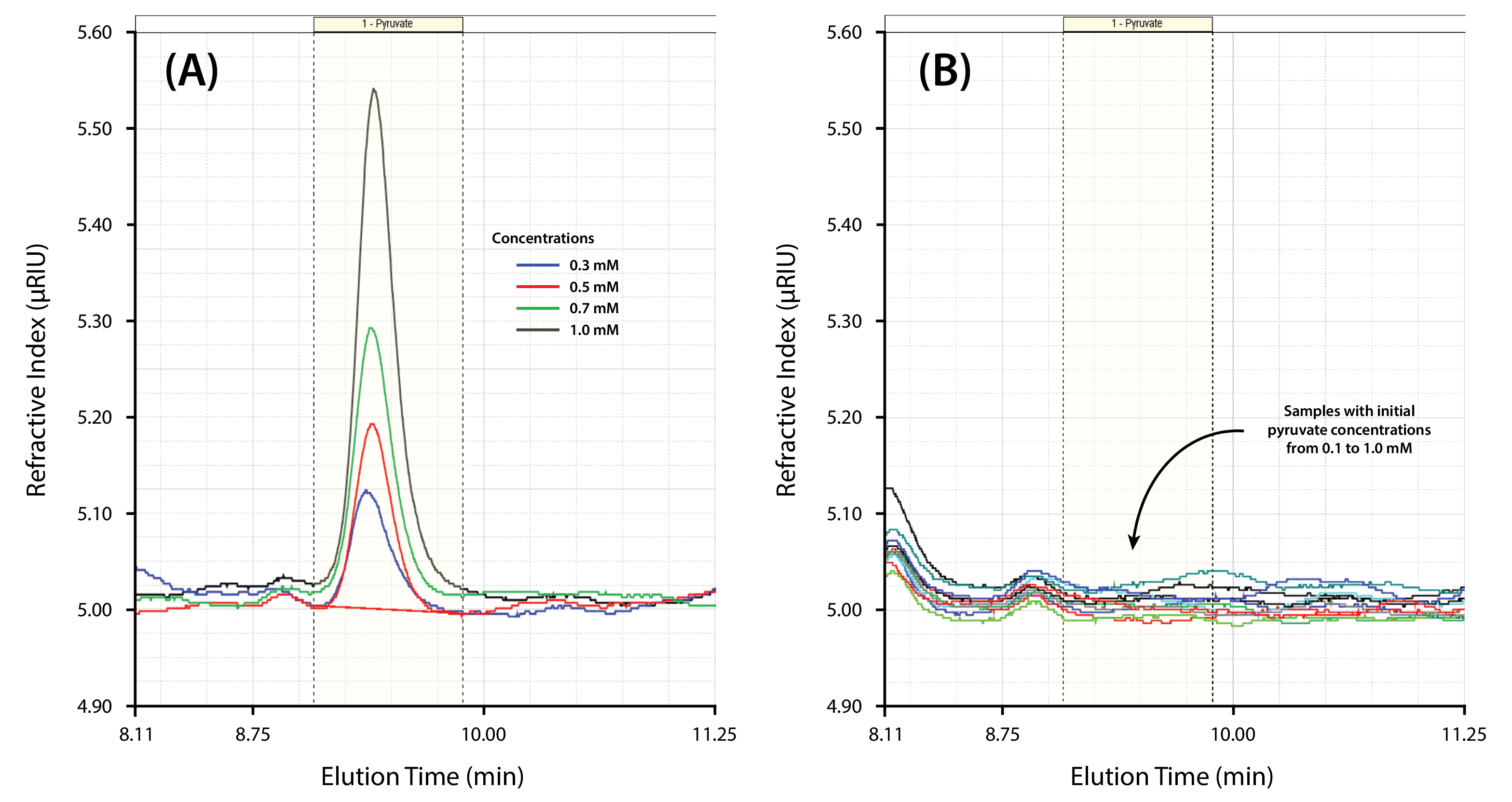


**Figure S4.** HPLC spectra showing (A) the pyruvate standards and (B) the pyruvate remained in the assay mixtures after transamination reactions. Complete depletion of pyruvate was observed in reactions with initial pyruvate concentrations from 0.1 to 1.0 mM, which are good representations of reaction conversions.


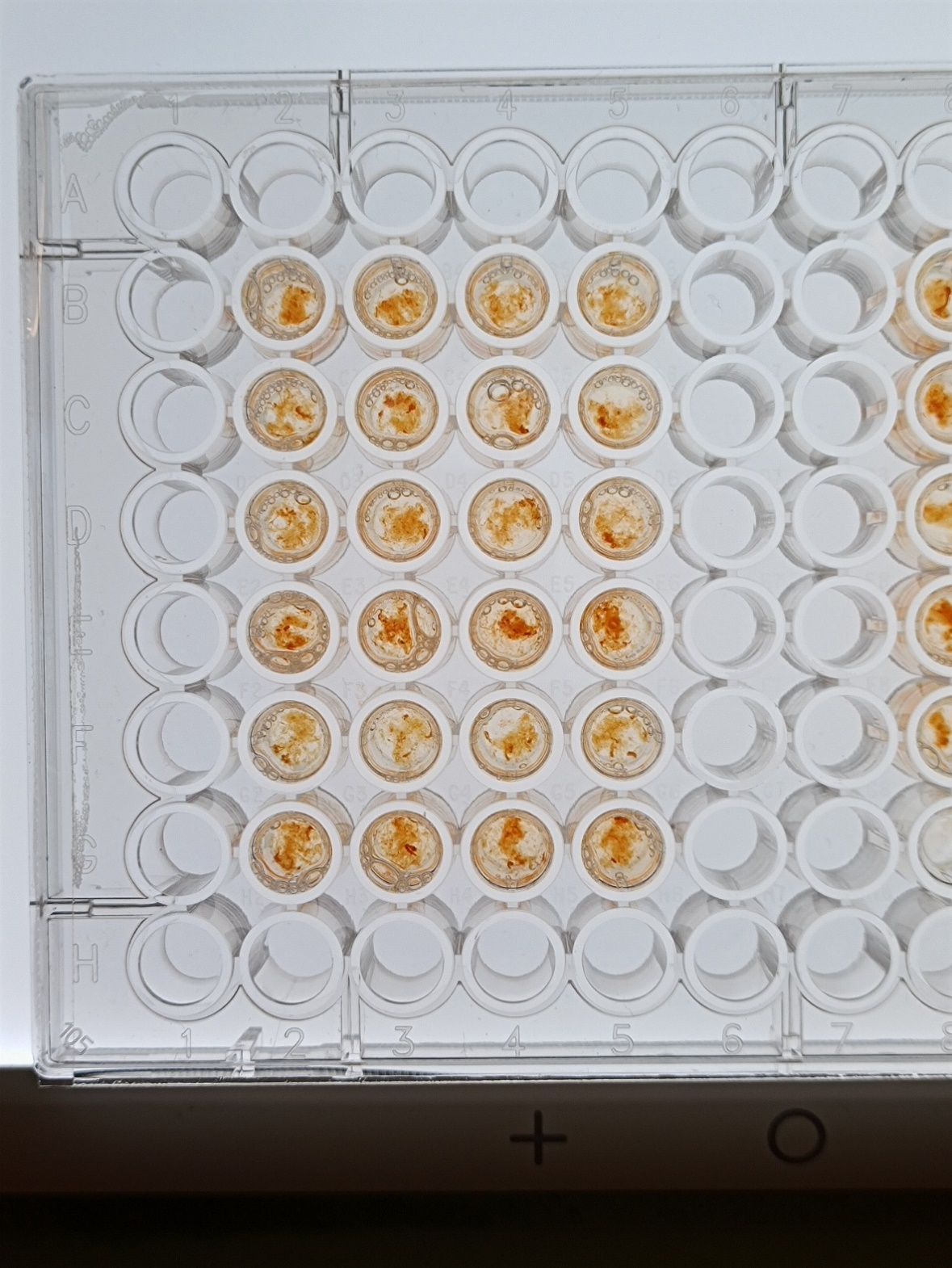

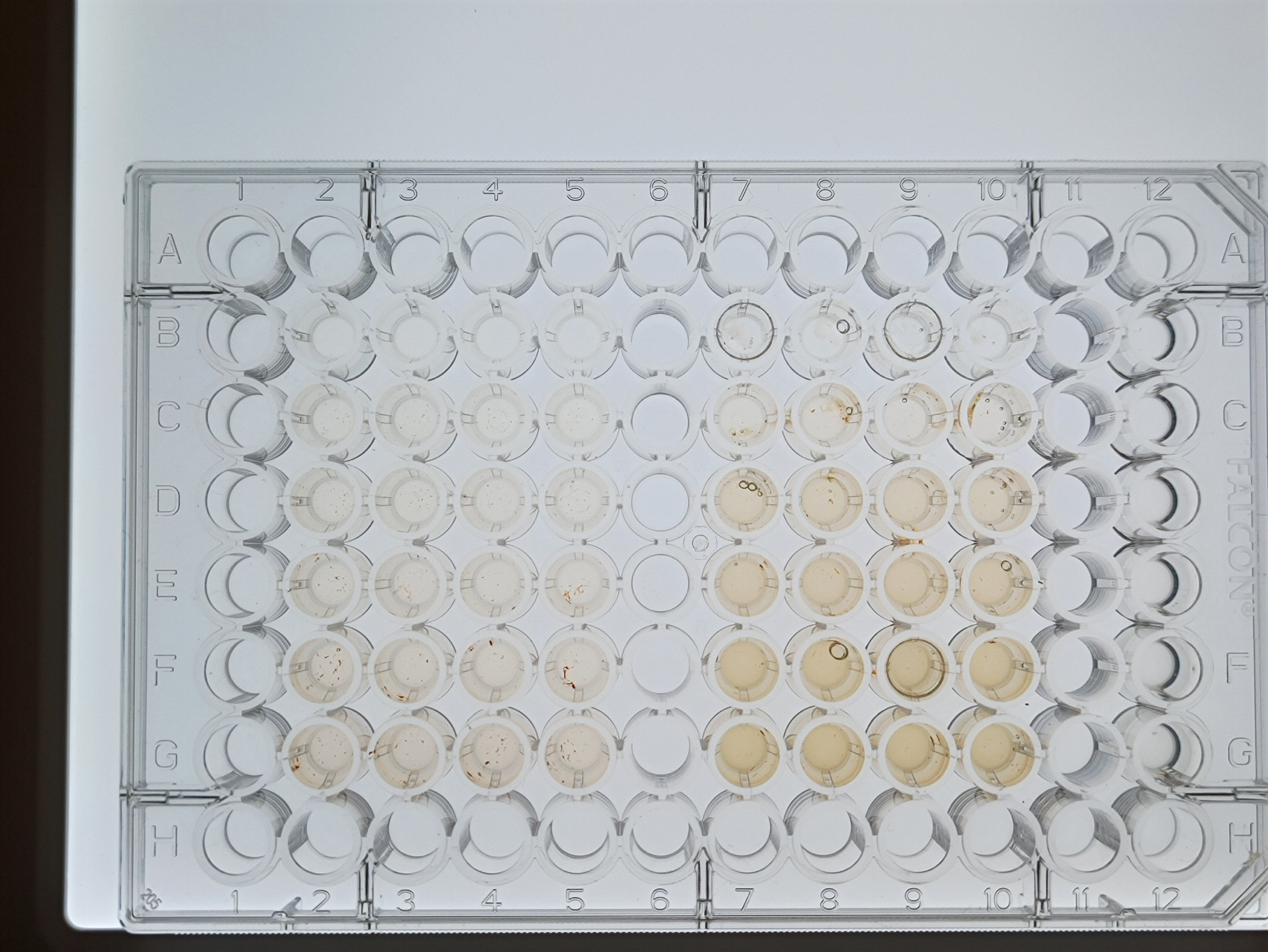


(B)

(A)

**Figure S5.** (A) Gel formation in galactomannan amination due to acetyl bond formations between oxidized intermediates. The reactions were conducted in one-pot following the standard Q-NPEA assay protocol. The red color of the gel came from the entrapped precipitates. (B) Galactomannan amination with reduced *Fgr*GaOx, catalase and HRP concentrations (0.2x of the original loadings). Compared to aminated galactomannan produced using the original *Fgr*GaOx loading, the products here showed more uniform properties and much less gel formation.


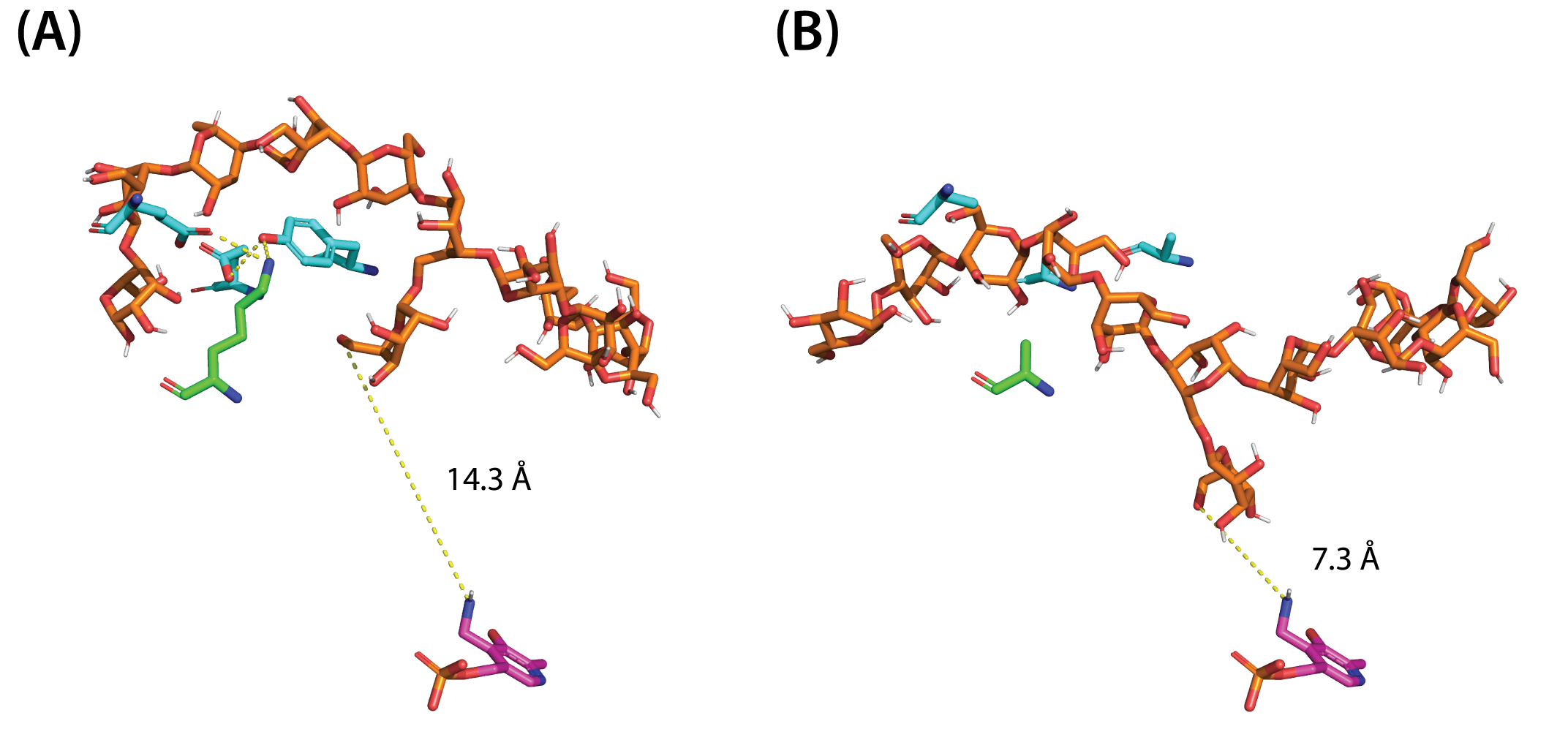


**(B)**

**(A)**

**Figure S6.** Docking simulation results showing the binding of oxidized galactomannan to the SpATA surface (A) before and (B) after alanine substitutions. The polysaccharide backbone binds closer to the SpATA surface after alanine substitutions and the distance between the oxidized galactose sidechains and the cofactor is considerably reduced. Amino acids with different colors are located on different polypeptide chains. Distances are measured from the carbonyl oxygen of the aldehyde on oxidized galactose sidechain to the amino group of the PMP cofactor. The reduced distance between reactive groups is likely correlated with increased rate of reaction.


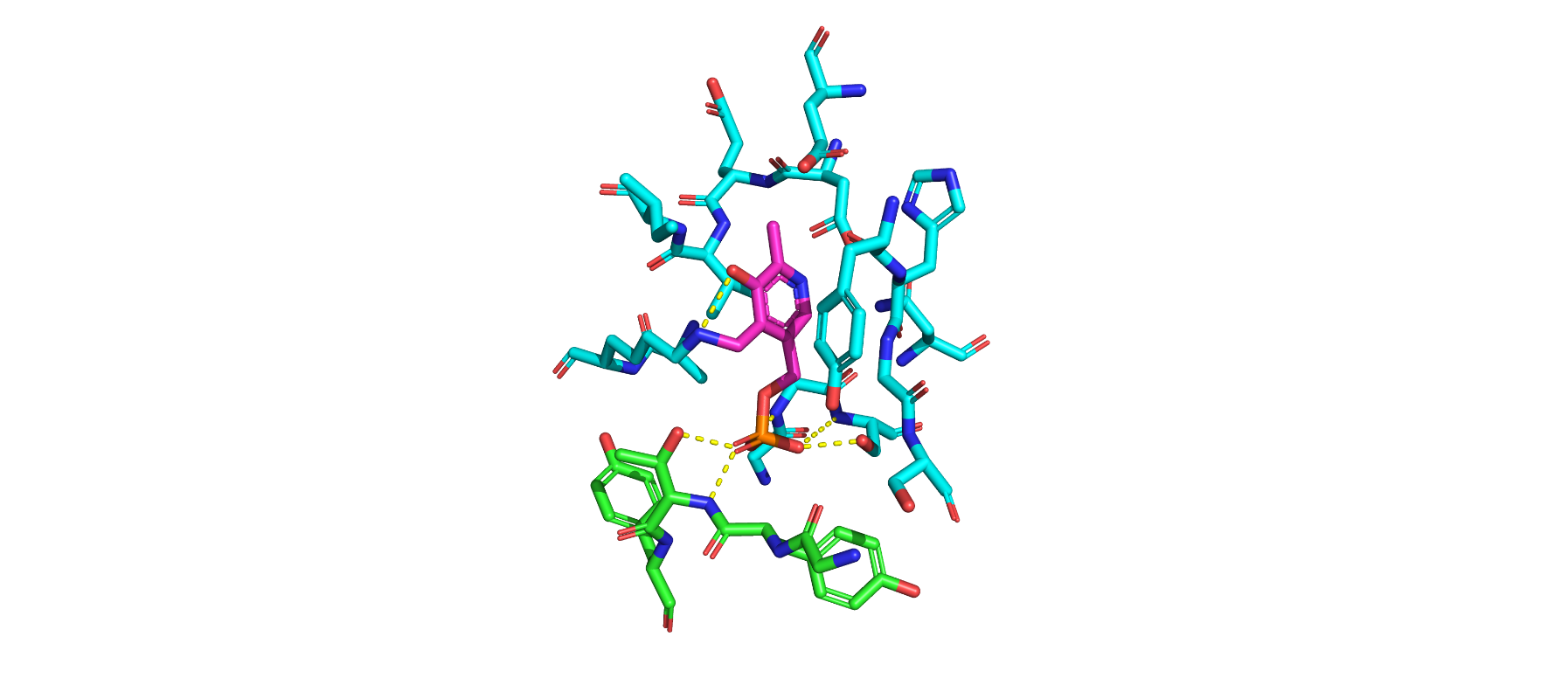

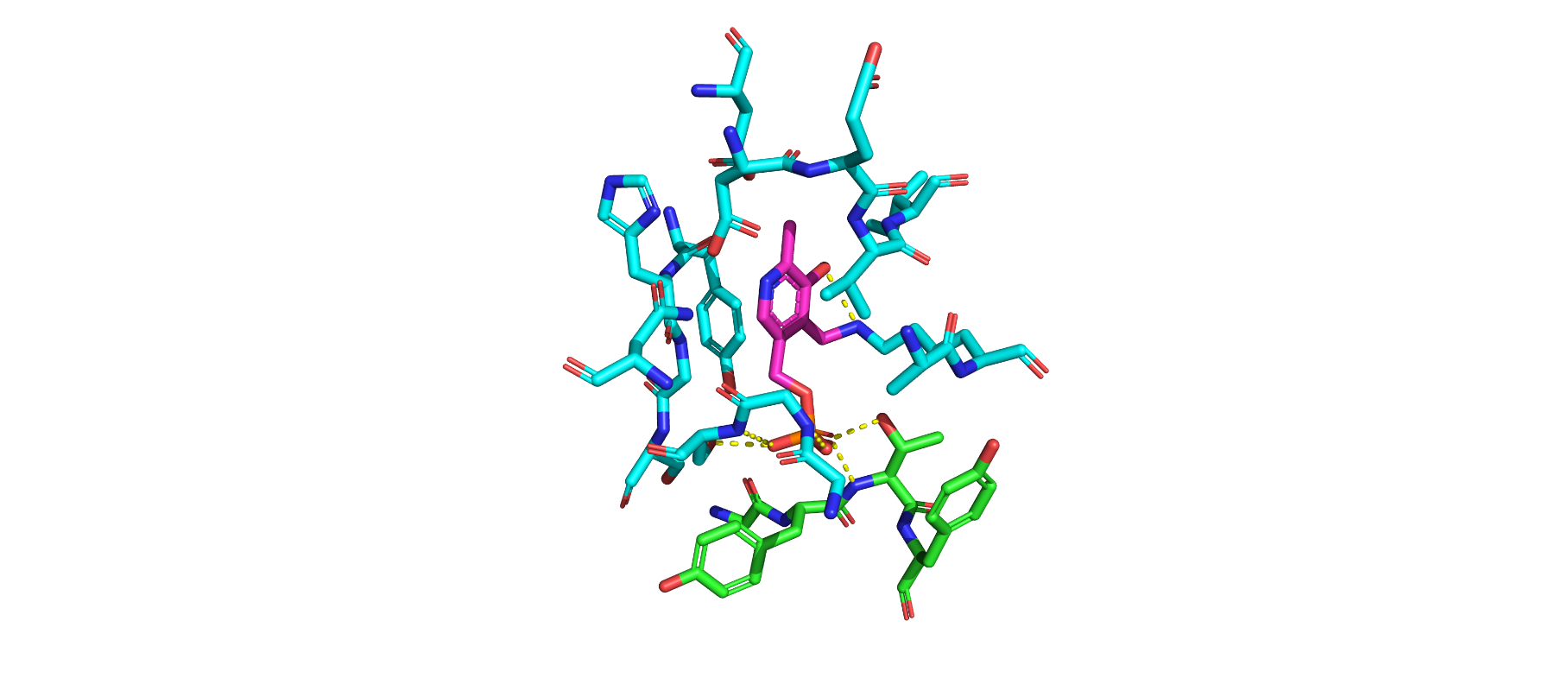


**Figure S7.** Active site of SpATA showing the PGBC hosting the PLP cofactor (magenta). The PLP binding pocket consists of amino acids from both monomers and is partially held together by polar interactions bridged by the PLP phosphate group. Amino acids having the same color are from the same chain.


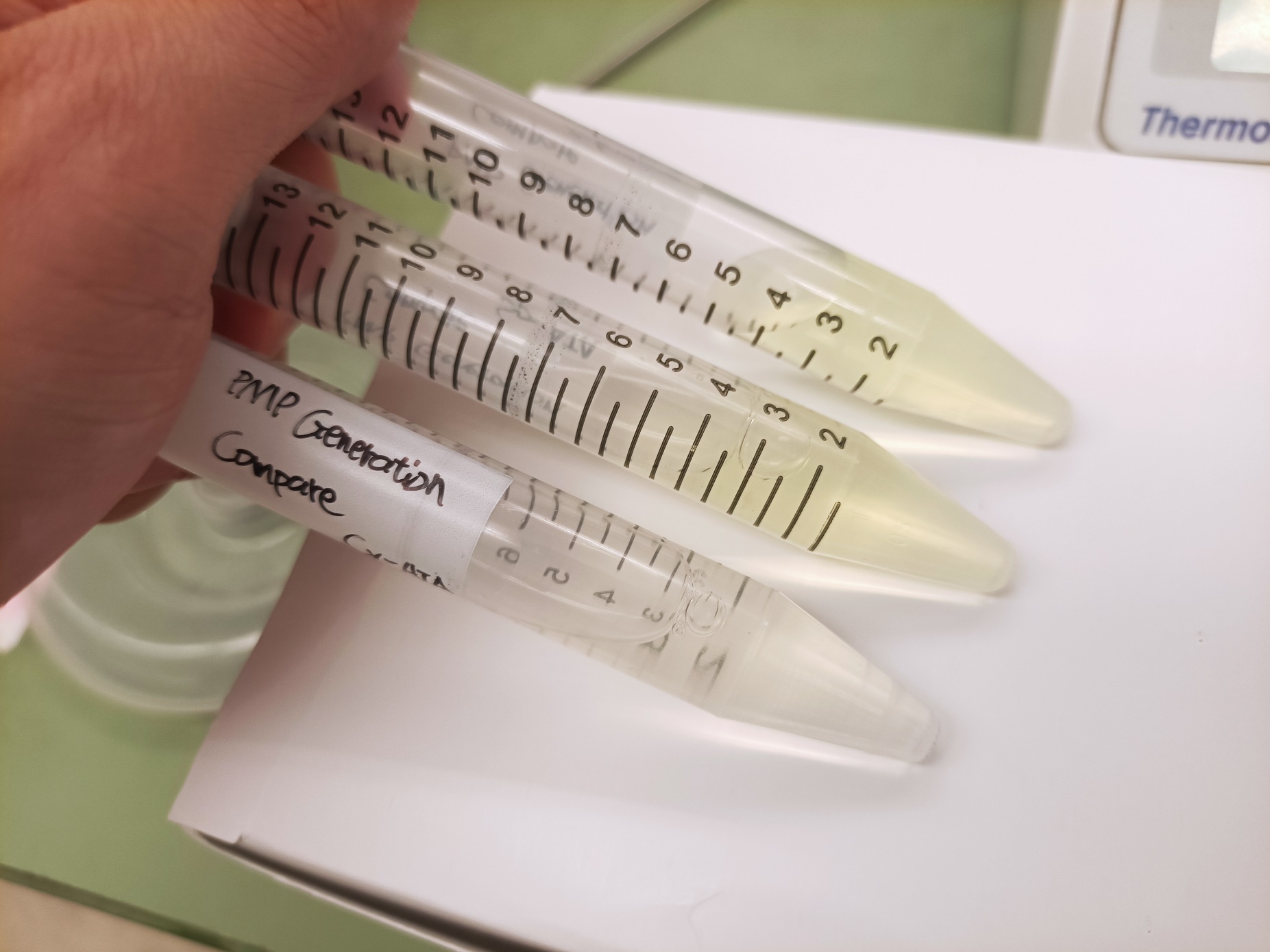


**SpATA Catalyzed Sample**

**CvATA Catalyzed Sample**

**No ATA Negative Control**

**Figure S8**. Discoloration of PLP in presence of (S)-1-PEA catalyzed by ATAs. Reaction mixtures (5 mL) comprised 1 mM PLP, 1 mM (S)-1-PEA and 0.15 mg/mL SpATA or CvATA in water. The reactions were performed in 15 mL centrifuge tubes and were incubated in a shaking incubator operating at 37 °C and 200 rpm for 48 h.


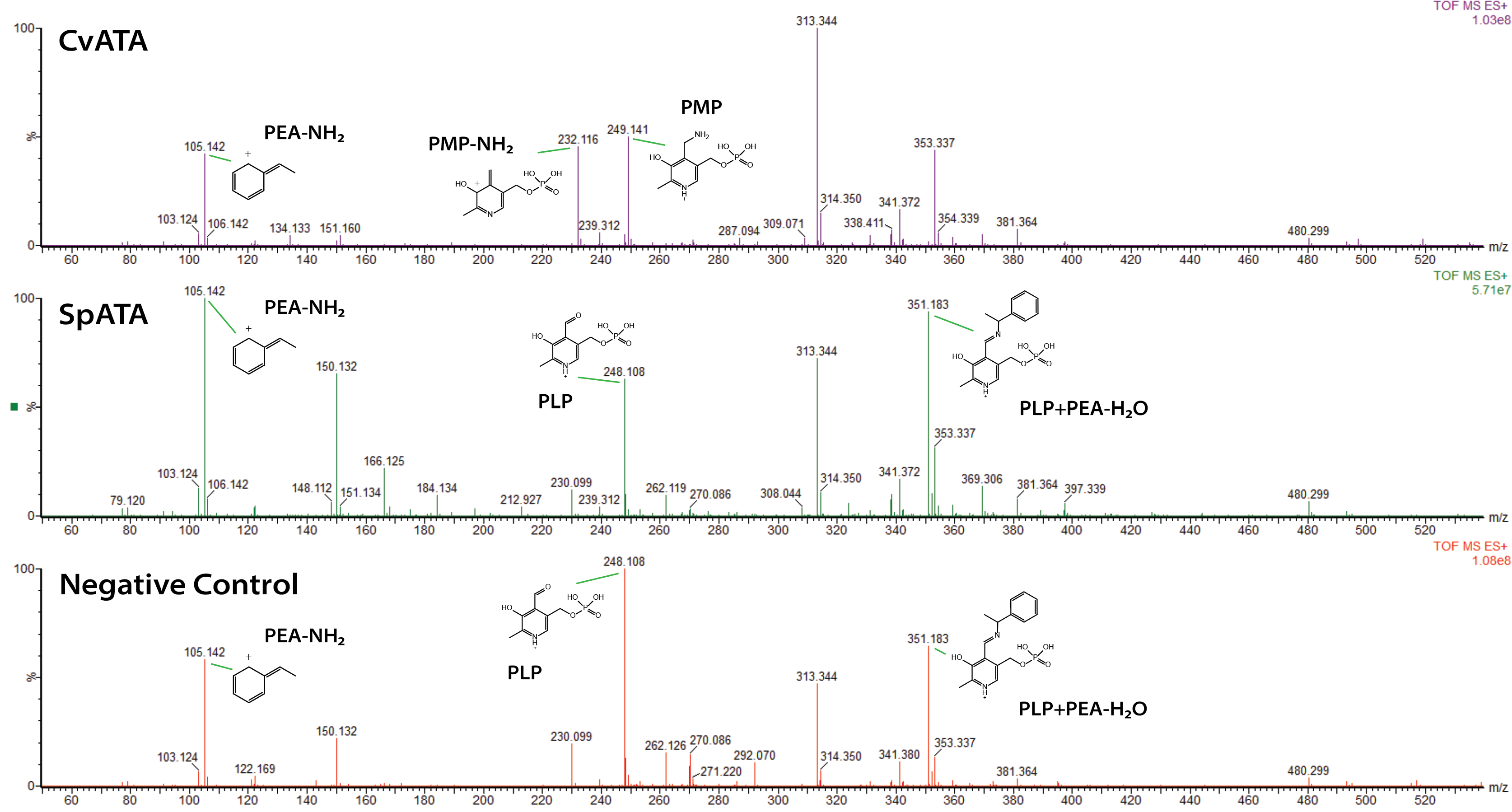


**Positive Ionization Mode**


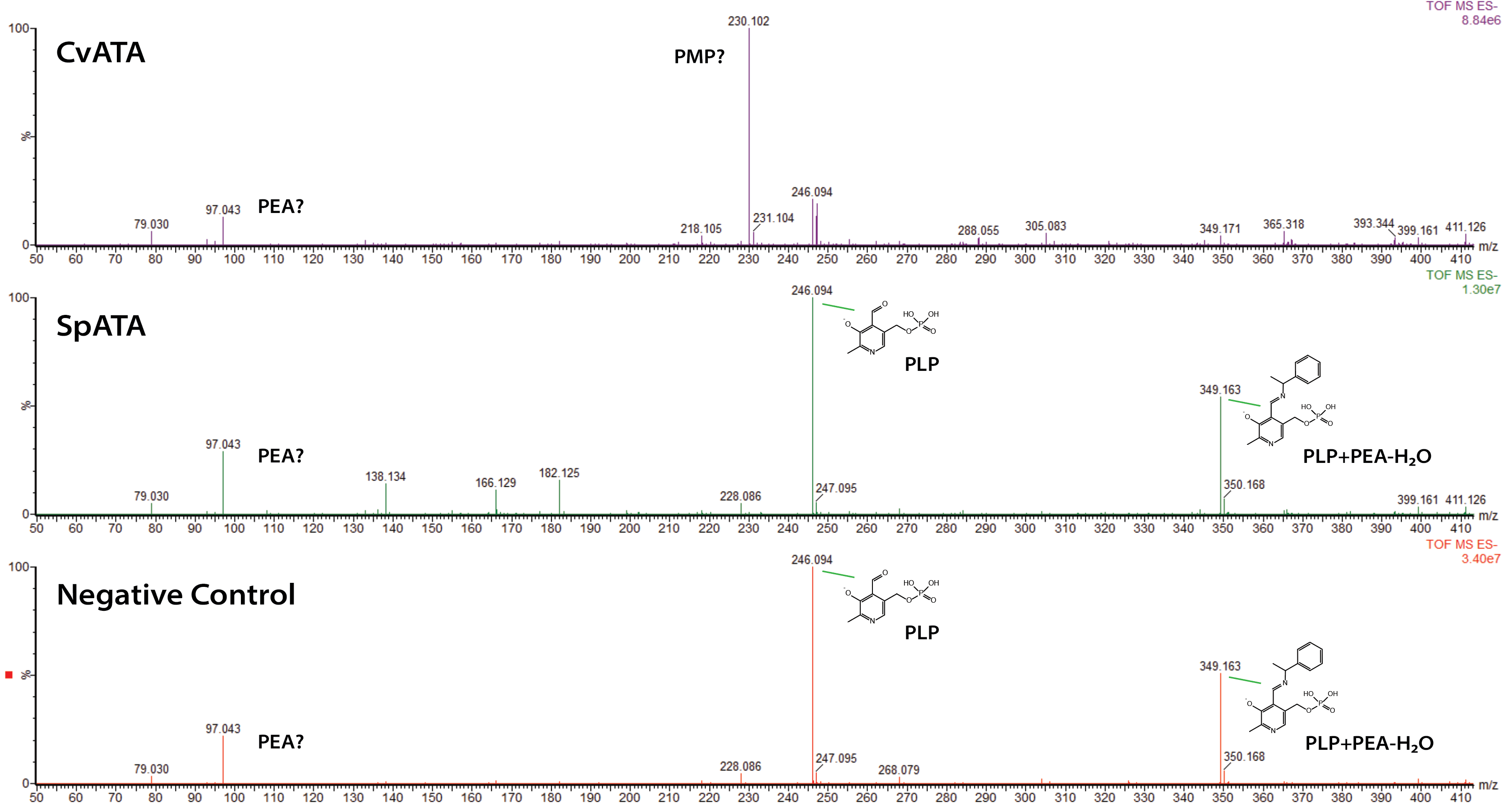


**Negative Ionization Mode**

**Figure S9.** The ESI-Q-TOF MS analysis on the reaction mixtures shown in Fig S7. The formations of PMP related compounds were detected in the CvATA catalyzed sample only. The composition of SpATA catalyzed samples largely resemble that of the negative control.

**Figure S10**. Initial rates of the wildtype SpATA and SpATA E407A mutant measured on pyruvate using the acetophenone assay. The large difference in initial rates at high pyruvate concentrations is attributed to the difference in active fractions of the two ATAs. An ATA loading of 300 ng was used in all reactions.


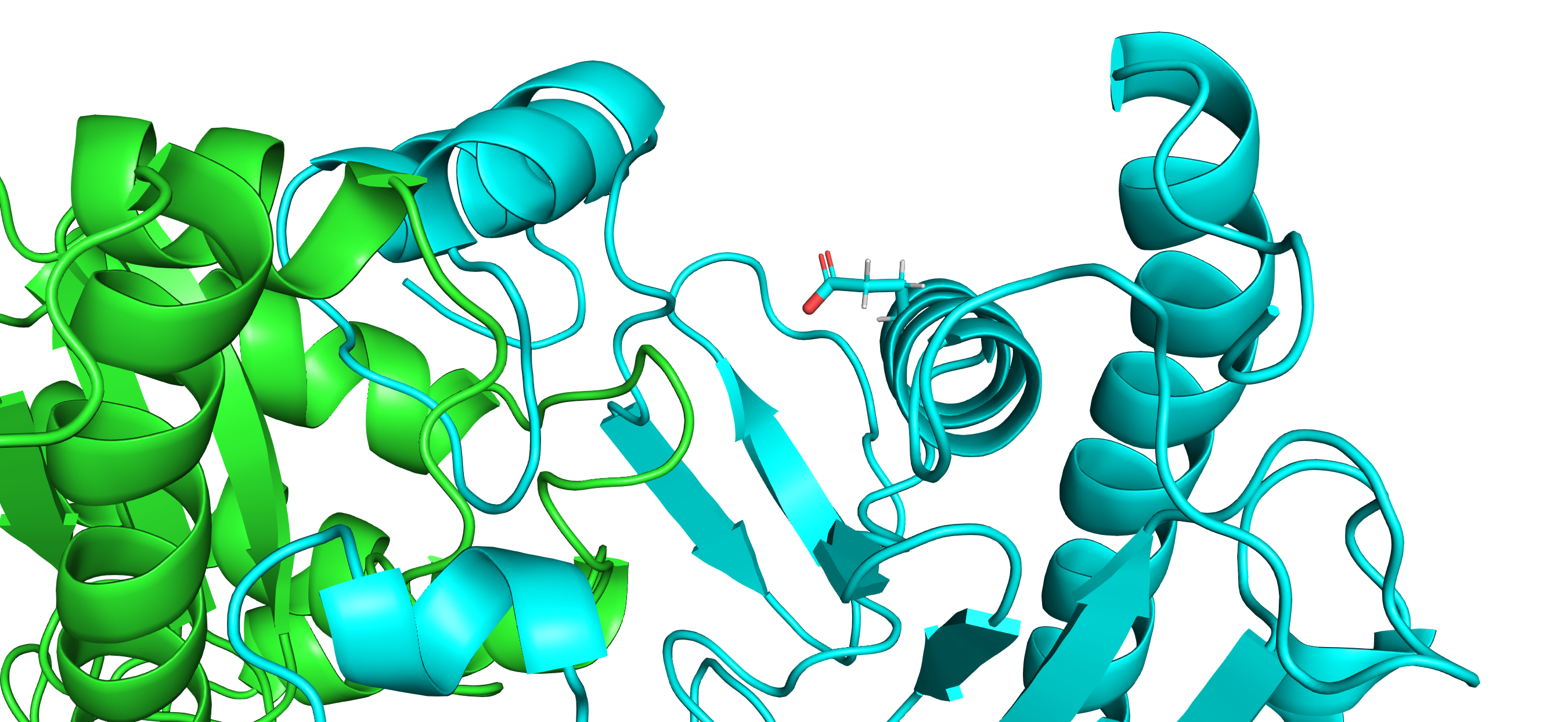


**Interfacial Loops**

**E407**

**Figure S11**. The location of E407A mutation near the interfacial loops of SpATA. The two colors indicate two different ATA monomers. The interfacial loops function as interlocks to prevent dimer dissociation and maintain active ATA structures.

**Figure S12**. Inhibition of FgrGaOx by aminated carbohydrates investigated using the ABTS assay and using C-2 glucosamine as the model animated product. As shown by the very similar absorbance readings measured from experimental groups with and without the addition of C-2 glucosamine, no significant inhibition of FgrGaOx was observed.

**Table S1.** A list of primers pairs used in the site-directed mutagenesis of SpATA engineering. Due to the close proximity of Y403 and E407, two versions of the Y403A primers were designed with and without the E407A mutation.

| Primer Pairs | Size | Sequences | T_m_ |
| --- | --- | --- | --- |
| E34A | 36 | 5’ – CAA TGC ACT GGG TGA AGC AGG CAC CCG TGT TAT TAC – 3’ | 66.9 °C |
|  |  | 5’ – GTA ATA ACA CGG GTG CCT GCT TCA CCC AGT GCA TTG – 3’ |  |
| K93A | 37 | 5’ – GTA TTA CAA CAC CTT TTT CGC AAC CAC GCA TGT TCC G – 3’ | 64.6 °C |
|  |  | 5’ – CGG AAC ATG CGT GGT TGC GAA AAA GGT GTT GTA ATA C– 3’ |  |
| Y403A (1) | 32 | 5’ – CGT GCA CGG CAG ATG GCG CCA ATC GTA CCC GG – 3’ | 72.3 °C |
|  |  | 5’ – CCG GGT ACG ATT GGC GCC ATC TGC CGT GCA CG – 3’ |  |
| Y403A (2) | 32 | 5’ – CGT TCA CGG CAG ATG GCG CCA ATC GTA CCC GG – 3’ | 70.7 °C |
|  |  | 5’ – CCG GGT ACG ATT GGC GCC ATC TGC CGT GAA CG – 3’ |  |
| E407A | 28 | 5’ – CGC GAA ACA GCG TGC ACG GCA GAT GTA G – 3’ | 66.6 °C |
|  |  | 5’ – CTA CAT CTG CCG TGC ACG CTG TTT CGC G – 3’ |  |
